# Spatiotemporal patterns and co-occurrence patterns of dissimilatory nitrate reduction to ammonium community in sediments of the Lancang River cascade reservoirs

**DOI:** 10.3389/fmicb.2024.1411753

**Published:** 2024-06-19

**Authors:** Bo Yuan, Mengjing Guo, Xiaode Zhou, Miaojie Li, Shuguang Xie

**Affiliations:** ^1^College of Geology and Environment, Xi’an University of Science and Technology, Xi’an, China; ^2^Faculty of Water Resources and Hydroelectric Engineering, Xi’an University of Technology, Xi’an, China; ^3^College of Environmental Sciences and Engineering, Peking University, Beijing, China

**Keywords:** DNRA, sediment, *nrfA* gene, cascade reservoirs, biogeographical pattern, Lancang River

## Abstract

Dissimilatory nitrate reduction to ammonium (DNRA) is an important nitrate reduction pathway in freshwater sediments. Many studies have focused on the DNRA process in various natural habitats. However, the joint operation of cascade reservoirs will affect the physical and chemical properties of sediments, which may change the DNRA process and bacterial community pattern in the surface sediments of cascade reservoirs. Our study was the first to investigate the spatiotemporal distribution patterns of potential DNRA rate, *nrfA* gene abundances, and DNRA bacterial community diversity in surface sediments of the Lancang River cascade reservoirs. The results of slurry incubation experiments combined with the ^15^N isotope tracer experiment ascertained that the potential rates of DNRA were 0.01–0.15 nmol-N cm^−3^ h^−1^, and qPCR results indicated that the abundance range of *nrfA* was 1.08 × 10^5^–2.51 × 10^6^ copies g^−1^ dry weight. High throughput sequencing of the *nrfA* gene revealed that the relative abundance of *Anaeromyxobacter* (4.52% on average), *Polyangium* (4.09%), *Archangium* (1.86%), *Geobacter* (1.34%), and *Lacunisphaera* (1.32%) were high. Pearson and RDA correlation analysis exhibited that *nrfA* gene abundance was positively correlated with altitude, pH, OC, and sand concentration. *Anaeromyxobacter* was positively correlated with reservoir age and DNRA potential rate. The deterministic environmental selection process plays a crucial role in the formation of the DNRA bacterial community. Network analysis displayed that the dominant DNRA genus was the key population of the DNRA microbial community in the sediments of Lancang River cascade reservoirs. This study reveals that the variation of DNRA bacterial activity and community structure is largely driven by the construction of cascade reservoirs, and provides a new idea for further understanding the characteristics of the DNRA community in the cascade reservoir ecosystem.

## 1 Introduction

Rivers play an irreplaceable role in connecting terrestrial and marine ecosystems (Aufdenkampe et al., 2011; Fan et al., 2015; Anderson et al., 2018). Previous studies have shown that almost two-thirds of the world’s major rivers (rivers with a length of more than 1,000 km) were controlled by anthropogenic activities such as damming (Wright and Minear, 2019; Wu et al., 2019). River damming, especially cascade damming, inevitably changes the hydrological rhythm, sediment transport process, and nutrients transport flux to the ocean (Dickson et al., 2012; Beusen et al., 2015; Brase et al., 2018; Chen et al., 2020c). Nitrogen (N) is the core element that controls the nutritional levels of the aquatic environment. Excessive N retention in water leads to the excessive consumption of dissolved oxygen. Simultaneously, the N removal process also involves N_2_O and other greenhouse gas emissions, which directly threaten water ecology and induce a series of global environmental problems (Zhang et al., 2015; Cheng et al., 2019).

The microbial-driven process of N transformation has been the focus of the researchers (Zhou et al., 2012; Yang et al., 2013; Yao et al., 2018; Zhang et al., 2018a; Wang et al., 2020b). Nitrate (NO_3_^−^-N) is the main “fixed” form of N in a freshwater environment, which is a key node in N assimilation and respiration pathways, and plays an important role in providing N and electron receptors. However, unlike denitrification and anammox processes that reduce nitrate or nitrite (NO_x_^−^-N) to N_2_/N_2_O in the atmosphere (Hou et al., 2013; Zou et al., 2018; Yin et al., 2020). In a long-neglected N conversion process, nitrate dissimilated to ammonium, i.e., denitrification and dissimilatory nitrate reduction to ammonium (DNRA), the fate and dynamics of primary productivity and N in water bodies may be affected in some specific water environments (Roberts et al., 2014; Wang et al., 2020a; Pandey et al., 2020; Hernandez-del Amo and Baneras, 2021). The DNRA process uses NO_x_^−^-N as the electron acceptor to oxidize NADH (i.e., nicotinamide adenine dinucleotide (NAD) + hydrogen (H)) as ammonium nitrogen (NH_4_^+^-N), which remains in the environment, providing the substrate for nitrification and anammox processes (Wang et al., 2020a; Broman et al., 2021). The DNRA process can be divided into two stages. The first stage is similar to the denitrification process, and nitrate dissimilated reductase (*narS*) reduces NO_3_^−^-N to NO_2_^−^-N. In the second stage, nitrite reductases (*nirS*) reduce NO_2_^−^ to NH_4_^+^. Currently, the NIR enzyme of DNRA exists in *Escherichia coli*, which is encoded by the *nrfA* gene, and its auxiliary group is the tetracyclic C complex (Tao and Wen, 2016). In addition, some autotrophic sulfur bacteria and anammox bacteria also have DNRA functions, but the specific mechanism remains unclear.

The driving factors and potential mechanisms of DNRA are determined by environmental factors. There are significant differences in the final products among different ecosystems (Luvizott et al., 2019; Zhao et al., 2020; Wang et al., 2020b). In general, environmental factors including organic carbon load, nitrate availability, electron donor/acceptor (C/N) ratio, sulfide concentration, soil sand content, pH, NO_3_^−^/NO_2_^−^ and temperature, control this competition (Nizzoli et al., 2010; Papaspyrou et al., 2014; Roberts et al., 2014; Li et al., 2017; Pang and Ji, 2019; Hellemann et al., 2020; Pan et al., 2020; Wang et al., 2020a; Broman et al., 2021). Several studies have shown that DNRA is dominant in electron-rich regions with low nitrate availability and high temperatures (Roberts et al., 2014; Li et al., 2017; Luvizott et al., 2019; Pang and Ji, 2019; Aalto et al., 2021). Many studies on DNRA have focused on marine, estuarine, and wetland sediments, natural lakes, heavily polluted rivers, small single reservoirs, sewage reactors, and soil (Li et al., 2017; Luvizott et al., 2019; Broman et al., 2021; Huang et al., 2021; Zhang et al., 2021b; Wang and Lin, 2023; Zhang et al., 2023). However, the relative contribution of environmental and biogeographical factors to the geographical patterns of the DNRA bacterial community in the cascade reservoir ecosystem formed by the development of large rivers is less known. The influence of biogeographical factors, such as geographic distance, is still poorly understood. Understanding the key environmental factors controlling the DNRA process is important for better prediction of N dynamics. Making up for this knowledge gap will help us better understand the community assembly mechanism of DNRA bacteria in sediment lotic habitats, and predict the potential response of DNRA community succession to anthropogenic disturbance environments.

The Lancang River (the upper reaches of the Lancang-Mekong River), is the largest international river in Asia. At present, cascade dams of the Lancang River are being constructed at an unprecedented speed, and their effects on fish, vegetation, and zooplankton are extremely complex and uncertain (Li et al., 2013; Fan et al., 2015; Dai et al., 2019; Han et al., 2019; Xu and Pittock, 2019). However, specific research on the effects of functional bacteria on the N transformation process in the sediments of cascade reservoirs is lacking. To the best of our knowledge, there is still relatively little research exploring DNRA bacteria in cascade reservoirs. This study aimed to (1) investigate the potential rate, abundance, and correlation of DNRA bacteria in the sediments of Lancang cascade reservoirs; (2) explore the composition and co-occurrence patterns of the DNRA community using high-throughput sequencing analysis; and (3) reveal the relationship between important environmental factors and the patterns of DNRA bacteria. Hypothetically, this study should help understand the N cycling process and microbial mechanisms in the Lancang cascade reservoirs. Our study also emphasizes dam engineering factors for the formation of DNRA bacteria in a freshwater ecosystem, which is an extension of a previous study on the role of sediment physicochemical factors (Nizzoli et al., 2010; Pang and Ji, 2019; Wang et al., 2020b). Our study has undeniably improved our understanding of the distribution and functional patterns of DNRA bacteria in large-scale cascade development in rivers.

## 2 Materials and methods

### 2.1 Study area and sample research topic

The Yunnan section of Lancang River enters the province from Weixi County, Yunnan Province, and enters Laos from Mengla County, with a total length of 1,240 km, a catchment area of 9.02 × 10^4^ km^2^, and an average annual runoff of 7.60 × 10^10^ m^3^ at the exit section. Approximately 85% of the annual precipitation is concentrated during the wet season (November–April) (Han et al., 2019; Luo et al., 2019; Xu and Pittock, 2019). By 2019, eleven cascade hydropower stations in the Yunnan section have completed impoundment. Due to the short operating time of the four newly built hydropower stations in the upstream section, the lack of reservoir operation data, and the instability of sediment. This study selected 7 cascade reservoirs in the middle and lower reaches of the river as the research objects. Supplementary Table S1 lists the engineering characteristics of seven cascade dams in the middle and lower reaches.

**Figure 1:**
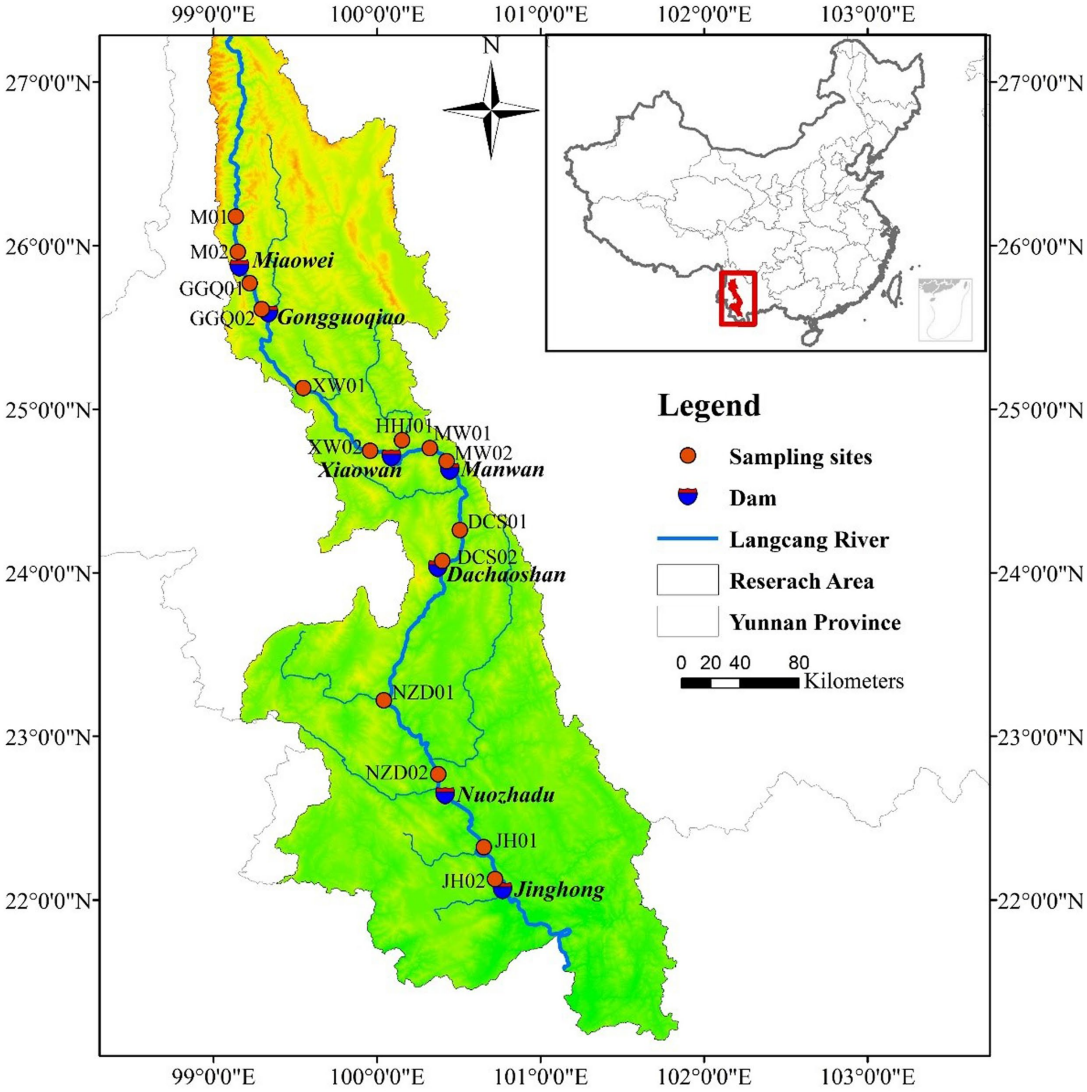
Sediment sampling sites in the cascade reservoirs of Lancang River.

To investigate the spatial and temporal distributions of DNRA bacterial communities in the Lancang River, two sampling campaigns (January 2019 and August 2019) were carried out. No extreme weather conditions occurred during the sampling period (Figure 1). Thirty surface sediment samples from the cascade reservoir were collected using a Peterson mud sampler. The longitude, latitude, and altitude information of each sample Research Topic site were determined using GPS (Supplementary Table S2). At all sampling sites, three surface sediment samples (0–10 cm) were homogenized and collected parallel to each other from three plots (2 m × 2 m). The samples for sediment slurry incubation experiments and environmental parameter analyses were placed in individual sterile plastic bags on ice (−4°C); the remaining subsample for molecular analysis was immediately sealed in a 50 mL polypropylene centrifuge tubes in liquid nitrogen (−80°C). All samples were immediately transported to the laboratory.

### 2.2 Sediment characteristic analysis

Before a chemical analysis, the sediment samples were freeze-dried, homogenized, and filtered through a 2.0 mm sieve. The pH value was analyzed using a Delta 320 pH Analyzer (Mettler Toledo, OH, United States) in a suspension of 5 g of sediment and 25 mL of 0.01 M calcium chloride. Sediment moisture was gravimetrically analyzed after desiccation for 72 h at 105°C. The grain size of the sediments was measured using a laser particle size analyzer (Mastersizer 2000; Malvern Co., United Kingdom). The total nitrogen (TN) content in the sediment was determined using the Kjeldahl method: 5 g samples and 20% KCl were added to the triangle bottle and filtered after concussion 0.5 h, and the concentrations of NH_4_^+^-N (using phenol-sodium hypochlorite spectrophotometry), NO_3_^−^-N (ultraviolet spectrophotometry), NO_2_^−^-N (diazo coupled spectrophotometry) were determined by using the filtrate. The total phosphorus (TP) content was measured using the molybdenum blue colorimetric method. The total organic carbon (Xu and Pittock) content was determined using a TOC analyzer (Elementar Liqui TOC II, Frankfurt, Germany). Three parallel samples were set up to determine the physical and chemical factors of all the samples.

### 2.3 Potential DNRA rate

The potential DNRA rate of the sediments in the cascade reservoirs was measured using the ^15^N isotope tracing method combined with sediment slurry incubation experiments. The basic method follows the previously reported potential denitrification and anammox rates (Hou et al., 2012; Yin et al., 2014). In brief, according to the sediment: water ratio of 1:5 (w/v), the collected fresh sediment samples and helium-purged overlying water from each site were prepared into slurries. Homogenized slurries were then transferred into 12 mL anaerobic serum bottles (Exetainer; Labco, HighWycombe, Buckinghamshire, United Kingdom) using a syringe under a fully aerated helium atmosphere. Samples were preincubated for 24 h in an incubator at *in situ* temperature to eliminate residual oxygen and NO_x_ (NO_3_^−^-N and NO_2_^−^-N). Subsequently, a standard solution of Na^15^NO_3_ (^15^N content of 99%, Cambridge Isotope Laboratories, Inc., Tewksbury, MA, United States) which was exposed to a helium atmosphere, was injected into the serum bottles with a syringe, and the final concentrations in the serum bottles were maintained at approximately 100 μM. Afterward, incubated sediment slurries were injected into 300 μL of 50% ZnCl_2_ solution to terminate the microbial activity and used as the initial time samples. The remaining samples were placed in the incubator under the same conditions as the shaking culture (200 rpm) for 8 h and then injected with 300 μL of 50% ZnCl_2_ solution to stop the reaction. Sediment slurries were then aerated with helium for 30 min to remove ^29^N_2_ and ^30^N_2_ from denitrification and anammox processes. Finally, 200 μL of hypobromite iodine solution was injected into the plasma bottle to oxidize ^15^NH_4_^+^ produced by DNRA into ^29^N_2_ and ^30^N_2_. The concentrations of ^15^N gases were measured by membrane inlet mass spectrometry (MIMS). The potential DNRA rate was calculated with the concentrations of the equation:


$$R_{\mathrm{DNRA}}=\frac{{\left[N15H_{4}^{+}\right]}_{\mathrm{final}}\times V-{\left[N15H_{4}^{+}\right]}_{\mathrm{initial}}\times V}{W\times t}$$

where ${\left[N15H_{4}^{+}\right]}_{\mathrm{initial}}$ and ${\left[N15H_{4}^{+}\right]}_{\mathrm{final}}$ (mmol N/L) are the ^15^NH_4_^+^ concentrations of the initial and final samples, respectively, *V* (L) is the volume of the incubation serum bottle; *W* (g) refers to the dry weight of the sediment, and *t* (h) refers to the incubation time.

### 2.4 DNA extraction, amplification, and high-throughput sequencing of *nrfA* gene

Genomic DNA (0.5 g) was extracted from the sediment samples using the PowerSoil^®^ DNA isolation kit (Mo Bio Laboratories, United States) according to the manufacturer’s instructions. The quantity and purity of the extracted DNA were examined using a Nanodrop 2.0 spectrophotometer (Thermo Fisher Scientific, CA, United States). The extracted DNA samples were sequenced using the MiSeq Illumina sequencing platform following the manufacturer’s guidelines. The partial sequence of the *nrfA* region of bacteria was amplified for high-throughput sequencing using the primers nrfAF2aw (CARTGYCAYGTBGARTA) and nrfAR1 (TWNGGCATRTGRCARTC) with a length of approximately 250 bp nucleotides (Welsh et al., 2014; Wang et al., 2020b). Triplicate amplifications from each sample were mixed for library preparation. A sequencing library was prepared using the TruSeq Nano DNA LT Library Prep Kit (Illumina). After the library was confirmed to be qualified, Shanghai Personal Biotechnology Co., Ltd. (Shanghai, China) was commissioned to perform double-terminal sequencing analysis using an Illumina MiSeq PE250 sequencing platform. All sequence read data were deposited in the NCBI Sequence Read Archive under BioProject accession number PRJNA560792.

### 2.5 Quantitative PCR of the *nrfA* gene

The abundance of the *nrfA* gene in each sediment sample was measured by quantitative PCR (qPCR) using the primer set nrfAF2aw/nrfAR1 (annealing at 55°C). Quantitative PCR was performed using the Bio-Rad CFX96 System (Bio-Rad Laboratories Inc., Hercules, CA, United States) and AceQ qPCR SYBR Green Master Mix quantitative PCR reagents. Plasmid standards containing a single copy of the *nrfA* gene were generated from environmental samples. Standard curves ranging from 10^3^ to 10^9^ gene copies/mL were obtained using serial dilutions of linearized plasmids (pGEM-T; Promega) containing the cloned *nrfA* gene amplified from environmental DNA. The amplification efficiency and coefficient of determination (*R*^2^ > 0.99) were 94.46% for the *nrfA* gene.

### 2.6 Bioinformatics and statistical analyses

Using QIIME 2 (version 2018.06) (Caporaso et al., 2010) and invoking the UCLUST sequence comparison tool (Edgar, 2013), the obtained sequences were merged, amplicon sequence variants (ASVs) were divided by 97% sequence similarity, and the most abundant sequence in each ASV was selected as the representative sequence. The representative ASV sequences were compared with the template sequence of the corresponding database to obtain the taxonomic information of each ASV. The alpha diversity indices of each sample and the distribution of DNRA bacteria in the 30 samples were determined using the vegan and ASV table packages. A heat map was constructed with the 50 most abundant genera using R software (version 3.5.1).

Pearson correlation analyses were conducted using SPSS Statistics 22.0 (IBM, United States) to evaluate the relationships among potential DNRA rates, environmental properties, and the abundance and diversity of the *nrfA* gene. The Kruskal–Wallis test was used to analyze differences in the abundance and alpha diversity of DNRA bacteria in sediments classified by seasonal and reservoir groups. A hierarchical cluster analysis was applied to investigate the similarities of DNRA bacterial community structures using the squared Euclidean distance and Ward’s linkage method. To identify the beta diversity of DNRA bacteria, a non-metric multidimensional scaling (NMDS) analysis based on the Bray–Curtis distance was performed using the vegan package in R software (v.3.5.1). To test the statistical significance of differences in DNRA bacterial community structures between classified sediment groups, analysis of similarity, permutational multivariate analysis of variance, and principal coordinate analysis were conducted on the Bray–Curtis dissimilarity of species using 999 permutations. To determine the influence of environmental and geographical factors on the DNRA bacterial community in the sediments of cascade reservoirs, the geographic distance between the sampling sites was calculated by considering the flow direction and river network length (Mao et al., 2019). Mantel and partial Mantel tests were performed in R using the vegan package based on the correlation between the matrices of DNRA bacterial community similarities (weighted UniFrac distances), geographical distances (cumulative dendritic distance), and environmental distances (Euclidian distance) (Chen et al., 2018; Li et al., 2019). Redundancy analysis (RDA) or canonical correspondence analysis was conducted using CANOCO (version 5.0) for Microsoft Windows (Mao et al., 2019; Lu et al., 2020).

Co-occurrence network analysis is a novel tool for investigating microbial community structures and internal interactions (Rottjers and Faust, 2018; Wang et al., 2020b). The bacterial co-occurrence patterns of DNRA bacteria in the different seasons were constructed using the “igraph,” “Hmisc” and “qvalue” libraries in R (v.3.4.2). The various roles of a certain node in the co-occurrence networks were identified using within-module connectivity (Zi) and among-module connectivity (Pi) at the threshold of 2.5 and 0.62, respectively. The network-level and node-level topological features of a network were calculated in the “igraph” package. Network graphs were visualized using Gephi 0.9.2^1^.

## 3 Results

### 3.1 Physicochemical properties of sediments in the cascade reservoirs

The physicochemical properties of the sediment samples are summarized in Supplementary Table S3. The pH of the sediments ranged from 6.99 to 8.44 and showed insignificant differences among the samples. The longitudinal spatial patterns of the pH were consistent between summer and winter. The water content of the sediments varied between 16.9 and 28.3%, with significant seasonal differences (*p* < 0.01). In contrast to pH and water content, nutrient-related physicochemical properties were observed in overall low to high gradients along the cascade reservoirs. Specifically, TOC content in sediments ranged from 9.51 to 20.96 g kg^−1^, with higher values in the reservoir and lake sections than those in the transition sections for most sampling sites. NH_4_^+^-N content in sediments dramatically increased from upstream to downstream, and varied between 12.7 and 33.0 mg kg^−1^, with higher values in summer than in winter. NO_3_^−^-N content ranged from 1.9 to 4.5 mg kg^−1^, and was significantly positively correlated with NH_4_^+^-N content (*p* < 0.01). The seasonal variations in most physicochemical properties were less drastic compared to reservoir heterogeneity and were less consistent along the cascade reservoir continuum. The geographical locations of the dams and the environmental status around the sampling sites varied for the different parameters, and the concentration levels differed between winter and summer.

### 3.2 Spatiotemporal patterns of *nrfA* gene abundance and potential DNRA rates

**Figure 2:**
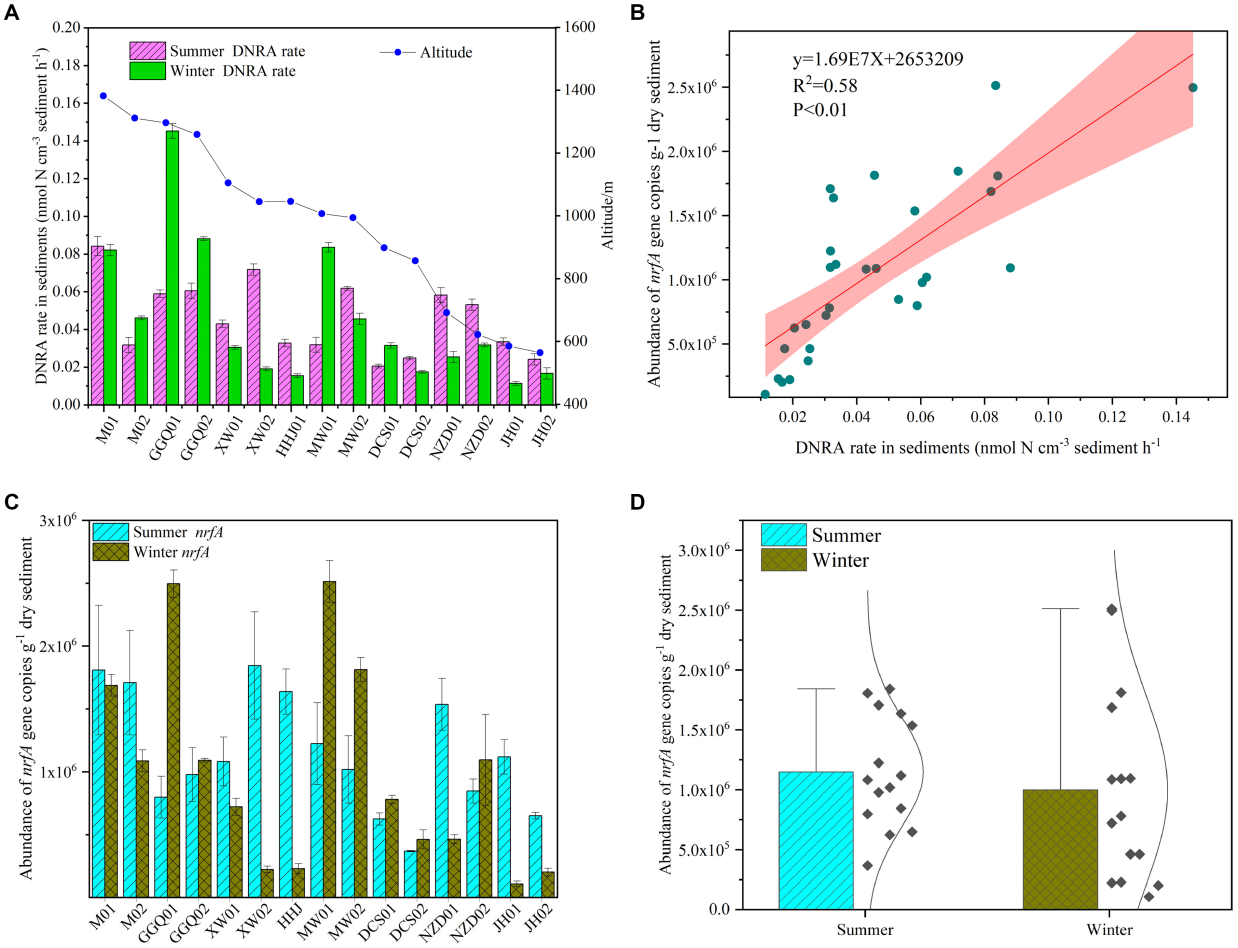
Seasonal variation of sediments DNRA bacterial abundance **(A,B)** and potential DNRA rates **(C,D)** in the cascade reservoirs.

The potential rates of DNRA in cascade reservoir sediments ranged from 0.01 to 0.15 nmol-N cm^−3^ h^−1^, and the potential rates were a little higher in the summer (0.06 ± 0.02 nmol-N cm^−3^ h^−1^) than in the winter (0.05 ± 0.03 nmol-N cm^−3^ h^−1^). The difference between the two seasons was statistically significant (*p* < 0.05). In addition, the variation in potential rates in the summer decreased gradually along the longitudinal direction of the reservoirs, and a similar trend was observed in the winter (Figure 2A). The highest values were observed at site M1 in the summer and GGQ1 in the winter, which were both upstream reservoirs. Interestingly, this pattern followed variations in *nrfA* gene abundance (Figure 2B). The *nrfA* gene abundance in cascade reservoir sediments was higher in the summer (1.15 ± 0.22 × 10^6^ copies g^−1^, *n* = 15) than in the winter (0.98 ± 0.08 × 10^6^ copies g^−1^, *n* = 15), and the differences between summer and winter were significant (*p* < 0.05), suggesting that bottom-water temperature (T) might be a key environmental factor that determined the abundance of the *nrfA* gene (Figures 2C,D). Meanwhile, a heterogeneous distribution of *nrfA gene* abundance was observed among the different sampling sites. In different seasons, the abundance of *nrfA* genes decreased gradually from the upstream reservoir to the downstream reservoir. A significant difference was observed between cascade dams (*p* < 0.05).

### 3.3 Spatiotemporal variations of DNRA bacteria richness and diversity

**Figure 3:**
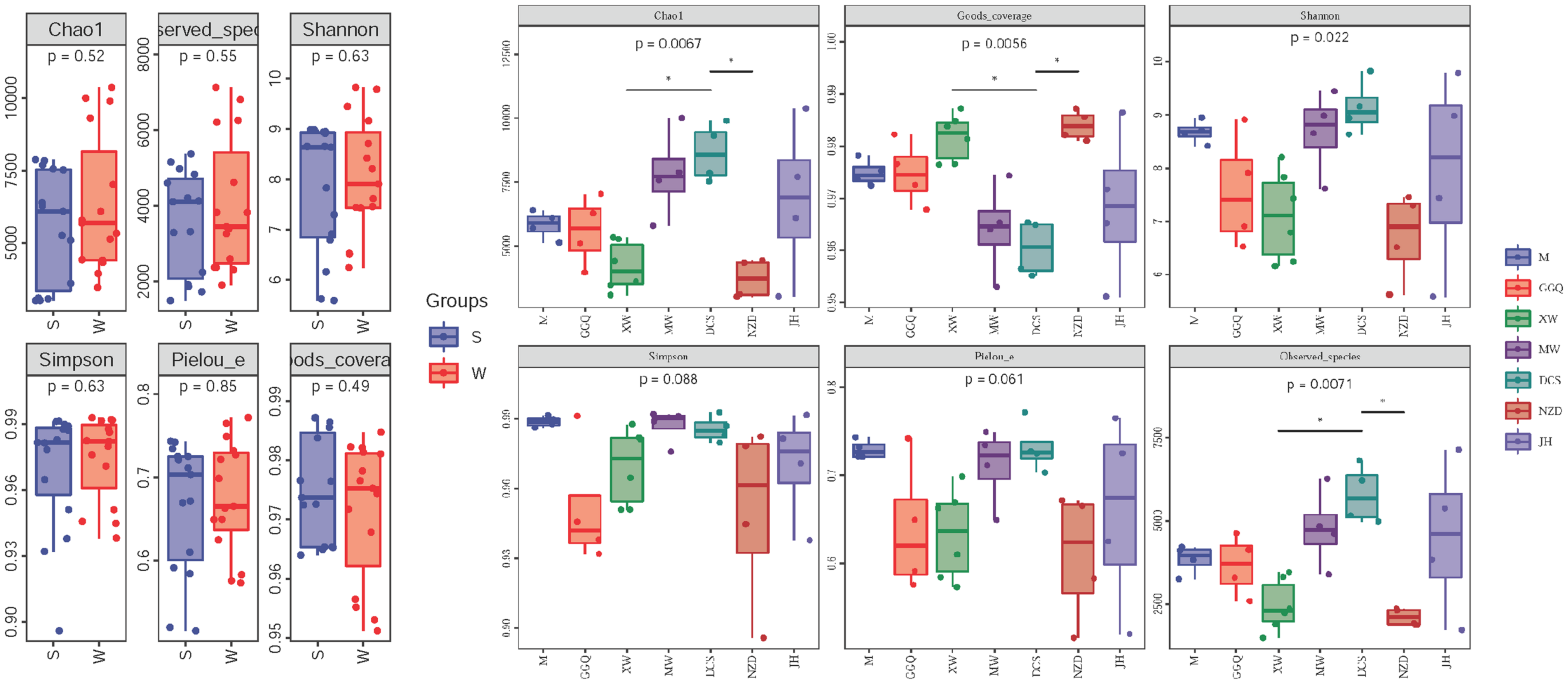
Comparison of diversity indices between seasons **(A)** and reservoirs **(B)**. *p*-values of t-tests between groups are indicated on the top of bar plots.

The statistics of the sequencing results for the sediment samples are summarized in Supplementary Table S4. The per-sample *nrfA* sequence count varied from 83,975 to 144,023. After the quality control processes (merge, filtration, and removal of chimeras), the unique per-sample *nrfA* sequence counts varied from 74,199 to 130,915. In all 30 sediment samples, 113,456 ASVs were obtained. The statistical data and rarefaction curves, as shown in Supplementary Figure S1, show the representativeness and fidelity of the rarefied data. The alpha diversity indices (Chao 1 estimator, Shannon index, good coverage, and observed species) of DNRA bacteria were significantly different among the cascade reservoirs in the spatial pattern (*p* < 0.05), but there was no significant difference between seasons (*p* > 0.05) (Figure 3A and Supplementary Table S5). The Chao1 estimator ranged from 3007.1 to 10364.4, the Shannon index, the Simpson index, the Observed species, and the Pielou’s evenness ranged from 5.58 to 9.82, 0.896 to 0.993, 1491.6 to 7,125, and 0.52–0.77, respectively. In addition, the observed species and Chao 1 estimators showed that the highest community richness occurred at site JH02 in the winter, whereas the lowest richness occurred at sites NZD01 and XW01 in the summer. Both the Shannon and Simpson indices at site DCS01 in the winter showed the highest diversity. Pielou’s evenness index was used to emphasize the evenness of the community; site DCS01 in the winter and site NZD02 in the summer had the highest and lowest community evenness, respectively.

### 3.4 Composition and distribution of DNRA community in the sediment of Lancang River

**Figure 4:**
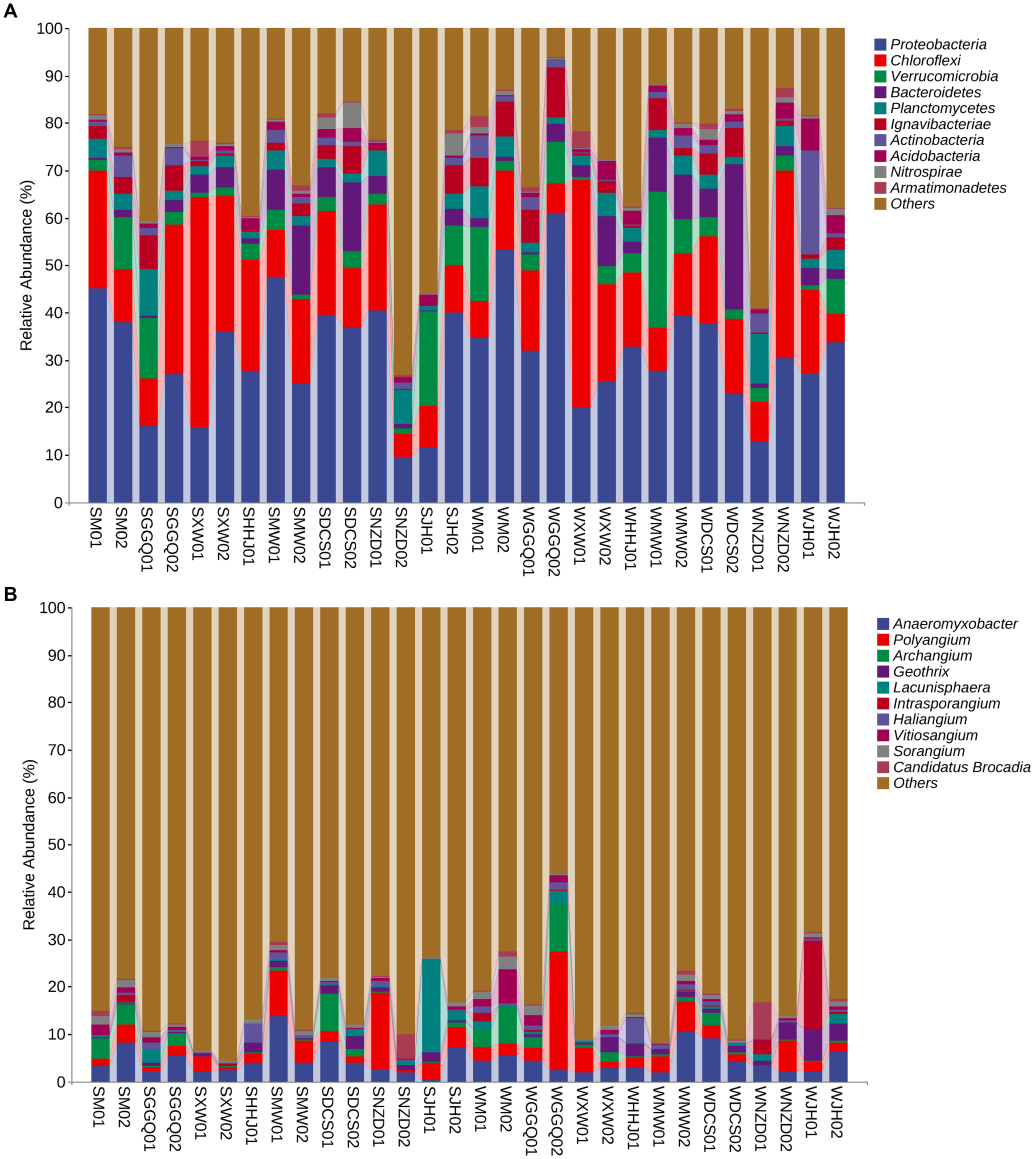
Taxonomic composition at the DNRA bacterial phylum level **(A)** and gene level **(B)** in the cascade reservoir sediments.

The relative abundances of DNRA communities at different classification levels were compared and tested between seasons and reservoirs. In general, seasonality in the relative abundance of dominant phyla or genera was weaker than their spatial variation across reservoirs. The dominant phyla (>1.0% relative abundance in each sample) are listed in Figure 4A. Proteobacteria, Chloroflexi, Verrucomicrobia, and Bacteroidetes were the four predominant phyla in all sediment samples, accounting for 9.56–61.17%, 4.87–48.38%, 0.82–28.73%, and 0.20–30.69%, respectively. A significant spatial pattern was also reflected at the genus level, although the seasonal variability was considerable, especially between deep-water reservoirs (XW and NZD) and other reservoirs in all seasons (Figure 4B). A genus-level analysis showed that the dominant genera were *Anaeromyxobacter* (4.52% on average), *Polyangium* (4.09%), *Archangium* (1.86%), *Geobacter* (1.34%), and *Lacunisphaera* (1.32%), whereas others were all less than 1.0%. However, no significant differences were observed between the two seasons.

**Figure 5:**
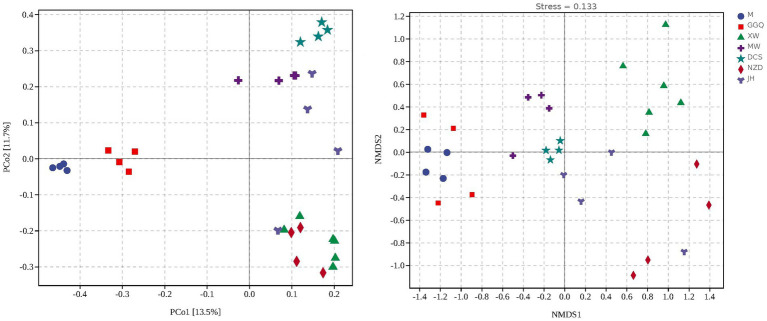
Principal coordinate analysis (PCoA) **(A)** and Non-metric multidimensional scaling analysis (NMDS) **(B)** illustrating sedimentary bacterial communities grouped reservoirs.

Based on the *nrfA* gene, hierarchical cluster (Supplementary Figure S2) and principal coordinate analyses (Figure 5A) showed that the samples at different sites from each reservoir in seasonal patterns clustered first (interpretation degree of 11.7%), while in the same season, reservoirs with the same regulation type preferred to cluster together (interpretation degree of 13.5% for the *nrfA* gene), suggesting a reservoir effect on dominant DNRA community composition. Similarly, with NMDS results rendered by groups of seasons and groups of reservoirs separately, the clustering effect of different seasons and reservoirs was shown in Figure 5B. An NMDS analysis showed that the communities of different sampling sites were separated, which was consistent with the variation in the relative abundance of dominant DNRA bacteria among the samples. In general, all samples were divided into four reservoir clusters: upstream reservoirs (M, GGQ), leading reservoirs (XW), midstream reservoirs (MW, DCS), and downstream reservoirs (NZD, JH). Furthermore, the correlation between different reservoir groups and distinct DNRA communities was confirmed using an analysis of similarity (*R* = 0.738, *p* = 0.001) (Supplementary Table S6). Interestingly, in contrast to the reservoir groups, seasonal groupings did not show a significant correlation with the DNRA communities (*R* = −0.022, *p* = 0.598). This indicated that the reservoir spatial variability (dam location and engineering characteristics) has a greater influence on the distribution of DNRA bacterial community than the temporal heterogeneity, which is consistent with the aforementioned result showing that the species diversity of different reservoirs was higher than that of the season.

### 3.5 Geographical and co-occurrence patterns of DNRA bacterial communities

Community dissimilarity in DNRA bacteria and geographical distance displayed a weak distance-decay relationship (*R* = −0.075, *p* > 0.01; Supplementary Figure S3). However, environmental distances exhibited a strong negative distance-decay relationship (*R* = −0.281, *p* < 0.001). The eight physicochemical properties of sediments obtained by the analysis involve the calculation of Euclidean environmental distance. Meanwhile, the slope of the distance-decay was −0.021, and the species turnover rate was 0.011 (*z*-value), which was involved in the calculation of the linearly modeled. Mantel tests were used to identify the relative contributions of environmental factors to the cumulative dendritic distance to DNRA bacterial community similarity. Interestingly, the Mantel and partial Mantel results revealed that the correlation coefficient between DNRA bacterial community similarity and environmental distance (*R* = −0.281, *p* < 0.001) was strong, whereas there was no obvious and significant correlation with cumulative dendritic distances (*R* = −0.075, *p* > 0.01). On balance, these analyses and results indicated that environmental drivers, rather than geographical drivers, determined the biogeographical patterns of DNRA bacterial communities in the Lancang River.

**Figure 6:**
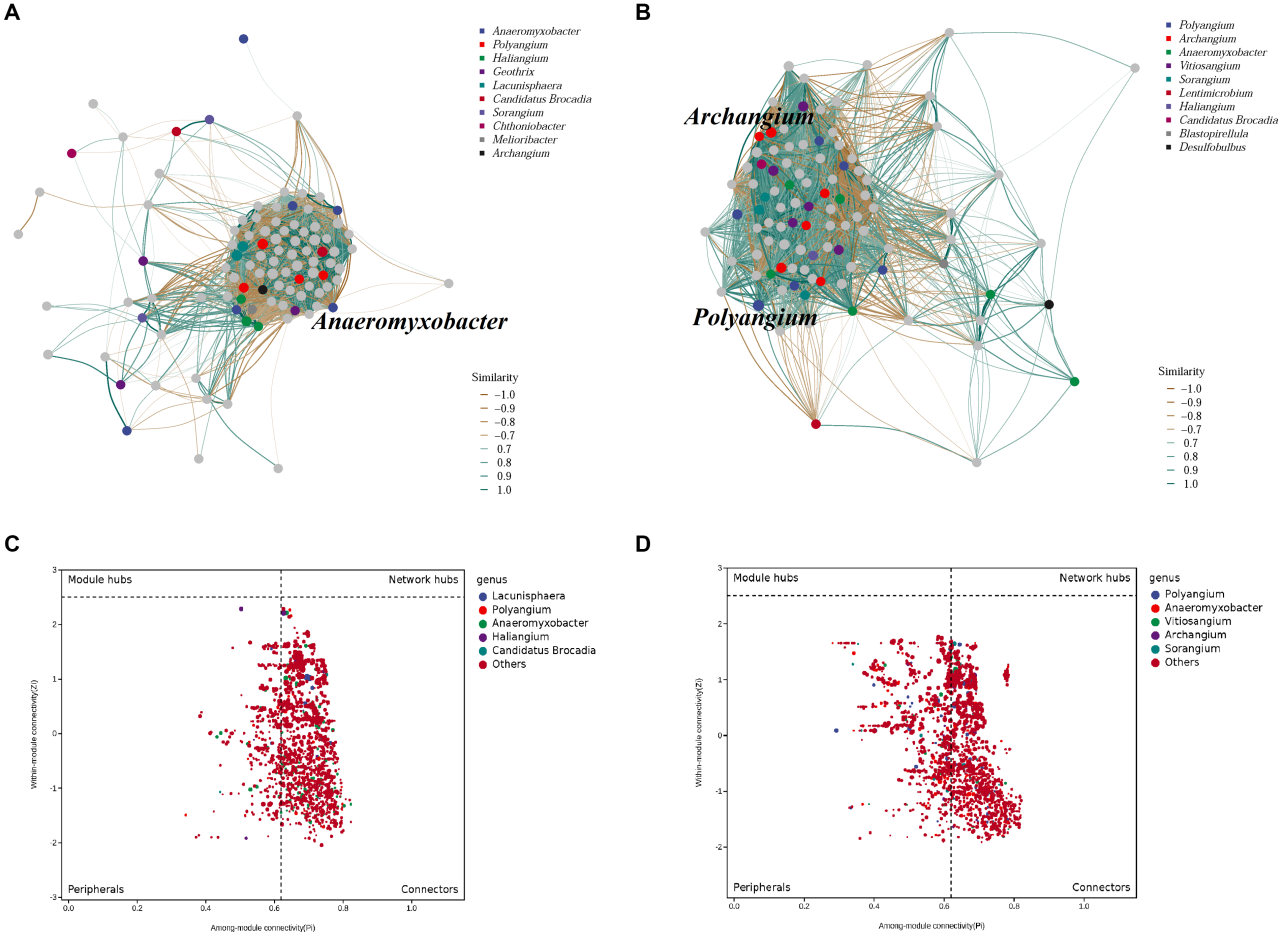
Characteristics of DNRA bacterial co-occurrence network. Correlation-based networks analysis showing the associations and putative connectors of DNRA bacteria.

The co-occurrence patterns of DNRA bacteria in the summer and winter in the cascade reservoirs were analyzed using a topological network analysis (Figure 6; Supplementary Figure S4). The network features showed that the summer sediments had lower density, transitivity, number of vertices, modularity, degree assortativity, and degree centralization than winter sediments. The summer network had 2,054 nodes connected by 273,088 edges with an *R*^2^ = 0.46; the genus *Haliangium* (ASV 331) in module 1 had the maximum degree of connection (Supplementary Table S7). The winter network had 2,360 nodes connected by 389,847 edges with an *R*^2^ = 0.57; the genus *Polyangium* (ASV 510) in module 1 had the maximum degree of connection. The results showed no significant difference in the composition and structure of the symbiotic network of the DNRA bacterial community between winter and summer, indicating that seasonal changes had no significant effect on the symbiotic network of DNRA bacteria in sediments. The nodes were classified as peripherals, connectors, module hubs, and network hubs according to the Zi and Pi values for the total network. The results of topological analyses have shown that 14.2% of the nodes can be classified as peripherals in the summer sediments, while 63.9% of the nodes belong to connectors in the winter sediments, and no nodes are in the module hub and network hub. The genus *Anaeromyxobacter* was the most connected in the summer network, but it was not in the Zi-Pi diagram of the module hubs; therefore, it could not be a key genus. The genera *Archangium* and *Polyangium* played a significant role between modules and within their respective modules during winter.

### 3.6 Relationships of potential DNRA rate, DNRA community patterns, and environmental drivers

The Pearson correlation analysis showed that the potential DNRA rate was positively correlated with NH_4_^+^-N (*R* = 0.602, *p* < 0.01), T (*R* = 0.497, *p* < 0.01), reservoir age (*R* = 0.436, *p* = 0.019), and water-depth (*R* = −0.429, *p* = 0.021) (Supplementary Table S8). The abundance of DNRA bacteria was significantly correlated with altitude (*R* = 0.433, *p* < 0.020), T (*R* = −0.423, *p* = 0.023), sands (*R* = 0.445, *p* < 0.016), and OC (*R* = −0.394, *p* < 0.035). Here, only the WRT of reservoirs exhibited significant influence on the alpha diversity of DNRA bacteria (*R* = −0.367, −0.381, 0.375, and −0.379, *p* < 0.05 in both cases), with the short WRT reservoirs harboring higher levels of alpha diversity than the long reservoirs. Moreover, the most dominant phylum, *Anaeromyxobacter*, had a positive and significant correlation with reservoir age (*R* = 0.504, *p* = 0.005) and the potential DNRA rate (*R* = 0.406, *p* = 0.029), whereas *Archangium* was significantly correlated with altitude (*R* = 0.461, *p* = 0.012), WRT (*R* = −0.480, *p* = 0.008) and pH (*R* = 0.378, *p* = 0.043). However, no positive correlation was noted between the DNRA rate and the dominant genus *Polyangium*. In contrast, genera with relatively small proportions, such as *Actinomyces*, *Syntrophus*, and the unclassified group, had positive and significant correlations with the DNRA rate (*p* < 0.05).

**Figure 7:**
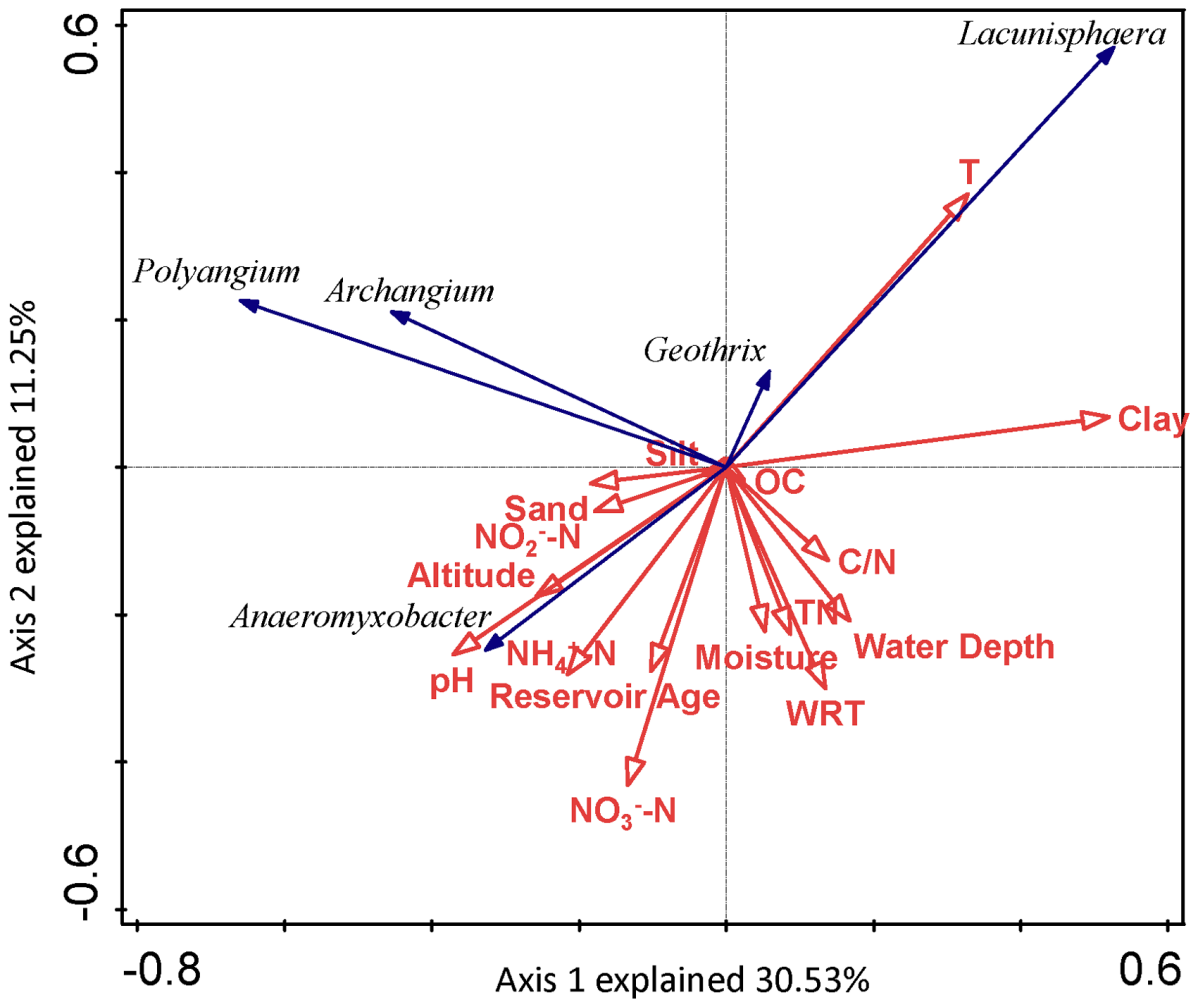
RDA ordination plot for the first two principal dimensions of DNRA bacterial communities and environmental factors.

The absolute values of ASVs that totaled more than 50 in sediments from the seven reservoirs were used as the species variables, and the environmental factors in the correlation analysis were used as the environmental variables (Figure 7). DCA was used to select the ordination type. As the maximum eigenvalue lengths for DNRA bacteria determined by DCA were 1.387 and 2.593, respectively, it was suitable to choose RDA to perform an ordination analysis. For DNRA bacteria, the first two ordination axes in the RDA explained 30.53 and 11.25% of the DNRA bacterial community composition, respectively. Factors including clay (*F* = 2.5, *p* = 0.038, 499 permutations) and T (*F* = 2.2, *p* = 0.041, 499 permutations) passed the Monte Carlo significance test, indicating that they were the most important environmental factors affecting the DNRA bacterial community structure in the sediments from the cascade reservoirs.

## 4 Discussion

### 4.1 Spatiotemporal patterns of DNRA bacterial abundance, potential rate, and diversity

As a dissimilatory reduction process, the DNRA is an important N removal/retention mechanism in freshwater sediments (Wang et al., 2020b; Pang et al., 2021) and other ecological environment systems (Supplementary Table S9), which is quite different from anammox and denitrification processes. NO_3_^−^-N can be transformed into N_2_ or N_2_O and removed permanently through denitrification and anammox (Pandey et al., 2020; Broman et al., 2021). Compared with previous studies on the eutrophic Pearl River estuary (Hu et al., 2021), eastern shallow lakes (Pang and Ji, 2019; Li et al., 2020), and Yellow River estuary (Bu et al., 2017), under the same primer conditions, the *nrfA* gene abundance determined in this study was lower, which may be related to the lower industrial development level of the Lancang River Basin, leading to a lower nutritional status of the river. Furthermore, the potential DNRA rates in our study were comparable to those of previous studies in some aquatic systems (Deng et al., 2015; Zhao et al., 2016; Pang and Ji, 2019), and exhibited a wide range. Affected by the continuous discharge operation of cascade reservoirs in the summer, the potential rate of the total sample showed a robust spatial pattern in summer. Our results suggested that NH_4_^+^-N may be the most critical environmental factor affecting the potential rate of DNRA (Supplementary Table S8). The Pearson analysis and RDA showed that the potential rate of DNRA in the Lancang cascade reservoirs was determined by T, NH_4_^+^-N in the sediments, water depth, and reservoir age. Therefore, the spatiotemporal patterns of DNRA in the sediments of cascade reservoirs may have been caused by spatial heterogeneity and nutrient concentration gradients at different sampling sites. Previous studies on temperate salt marshes and estuarine sediments have shown that high temperatures are beneficial for the potential rate of DNRA (King and Nedwell, 1984; Deng et al., 2015; Hu et al., 2021). This observation is consistent with our finding that the DNRA potential rate of cascade reservoirs in the summer is higher than that in the winter, and the DNRA potential rate of downstream reservoirs is the highest. Although the rate of DNRA is positively correlated with NH_4_^+^-N, it does not mean that the generation of NH_4_^+^-N is entirely generated by the process of nitrate dissimilatory reduction. The sources of NH_4_^+^-N in sediments may also include other processes such as organic matter mineralization and biological nitrogen fixation. DNRA is not only an important pathway for NH_4_^+^-N generation in sediments but also plays an important regulatory role in sediment N cycling and water N dynamics by affecting NH_4_^+^-N flux and mineralization processes (Bu et al., 2017; Wang et al., 2020b). Therefore, when studying this relationship, it is necessary to comprehensively consider the influence of multiple factors.

Interestingly, our results showed that the water depth and reservoir age showed a significant response relationship. A deeper water depth may provide a suitable anoxic environment for denitrification, thus enhancing denitrification, but inhibiting nitrate metabolism pathways, such as the DNRA process. The longer the reservoir age, the more abundant the labile carbon content in the sediment, providing sufficient electron donors for the DNRA process. Generally, the DNRA process is more likely to occur in environments lacking N sources and rich and unstable carbon sources. The effects of temperature and organic matter (carbon source) on the potential activities of DNRA are also controversial, indicating the complexity of microbial responses to climate warming and human activities. DNRA communities in different environments may have different ecophysiologies and habitat adaptabilities (Dang and Chen, 2017). For example, the microbial community in a temperate estuarine environment with clear seasonal and nutrient spatial gradients has experienced eutrophication and pollution, which may have adapted to different factors. This implies that in some freshwater environments, in addition to temperature and carbon sources, variations in environmental conditions may be the most important driver controlling the potential rate of DNRA.

In the cascade reservoirs, we found significant differences in α-diversity and abundance of different regulating types of reservoirs. There are many possible reasons for these results, but our research has shown that compared to other physicochemical characteristics, the variation in water temperature at the bottom of the reservoir has the most direct impact, which was similar to the changes in thermal stratification caused by different reservoir regulation rules. Compared with runoff hydropower dams, reservoirs with higher regulation performance (XW and NZD) have obvious thermal stratification, which significantly affects the water temperature of the overlying water and sediment at the bottom of the reservoir, thereby affecting the richness and diversity of DNRA bacteria in the sediment. DNRA bacteria are typical psychrotrophic and mesophilic bacteria, that are very sensitive to temperature and biomass production, and the individual development rate usually decreases with an increase in temperature (King and Nedwell, 1984; Brown et al., 2004). High temperatures are beneficial for the occurrence of DNRA in the sediments of temperate estuaries (King and Nedwell, 1984). Therefore, when there was no significant difference in nutrients between the two groups, the thermal stratification reservoir with a lower bottom water temperature could harbor a lower abundance of DNRA bacteria. However, previous studies on the incubation of sediments in the Pearl River Estuary showed that the ammonium production rate of DNRA bacteria in this area was the highest at 30°C, and then decreased with an increase in temperature (Tao and Wen, 2016; Hu et al., 2021).

Based on the deep theory of ecological metabolism (Comte and del Giorgio) revealed that the logarithmic transformation of bacterial α-diversity is a linear function of the reciprocal of absolute temperature, which increases with temperature (Brown et al., 2004). Moreover, the α-diversity of bacteria can grow rapidly with increasing temperature in the middle and mesophilic ranges (Tao and Wen, 2016; Chen et al., 2019). This inevitably leads to a low α-diversity of DNRA bacteria in a reservoir with high regulation. Additionally, soil from terrestrial ecosystems contains many types of DNRA bacteria, which are carried by water flow and suspended in sediment, showing significant spatio-temporal heterogeneity (Li et al., 2017; Pandey et al., 2020). The deposition of suspended sediment will lead to the encounter of the microbial community between the suspended sediment and riverbed sediment (the aggregation of the microbial community) (Rillig et al., 2015), which may affect the spatial pattern of DNRA bacteria.

### 4.2 Nutrients and dam engineering factors shaping spatiotemporal patterns of DNRA bacteria

The effect of cascade hydropower dams on the river ecosystem has always been a concern to the public (Wen et al., 2018; Wu et al., 2018; Chen et al., 2020a); however, there is a lack of understanding of the effects of N cycle processes. This study reported the community structure of the *nrfA* gene in the sediments of cascade reservoirs, which is distinct from that in estuarine sediments (Bu et al., 2017; Hu et al., 2021). The spatio-temporal pattern of the DNRA bacterial community was observed along the cascade reservoirs at the phylum and genus levels (Figure 4). Our result showed that Proteobacteria was the most abundant phylum of DNRA bacteria. Notably, at the phylum level, the relative proportion of Verrucomicrobia at the WMW01 site was more than seven times that of other sites, reaching 28.7%. In another typical anammox bacterium, *Planctomycetes*, the relative abundances of WNZD01 and SGGQ01 were approximately two or three times higher than those at other sampling sites, reaching 10.6 and 9.8%, respectively. *Planctomycetes* have been found in many habitats, including marine, freshwater, soil, and extreme habitats (Schlesner, 1994), which show strong competitiveness in the removal of SOC and ammonium (Liu et al., 2018a). Our results on DNRA composition at the genus level were mostly consistent with those of other studies in eutrophic lakes and estuaries (Roberts et al., 2014; Decleyre et al., 2015; Deng et al., 2015; Hardison et al., 2015; Domangue and Mortazavi, 2018; Pang and Ji, 2019; Pang et al., 2021). *Anaeromyxobacter* belonged to the class *Deltaproteobacteria* and was the dominant genus of DNRA. *Anaeromyxobacter*, a group of respiratory bacteria, can mediate both the reduction of Fe oxides and ammonium oxidation in some anoxic environments (Li et al., 2015b; Pandey et al., 2020; Liu et al., 2021). *Polyangium* was the most dominant group in the biofilm due to the specificity of habitat environments, such as low oxygen concentrations and low organic content (Liu et al., 2021). The relative abundance of DNRA bacteria in different reservoirs was obvious, and the variation of these genera may be attributed to the changes in physicochemical, hydrodynamic, and biological characteristics caused by damming in different reservoirs.

However, it must be acknowledged that this study mainly focuses on the relationship between DNRA activity and identified microbial species, while ignoring the majority of undetermined species in the DNRA community. Therefore, this method may have certain limitations. The DNRA process is a complex biochemical reaction involving multiple microorganisms and their interactions. If we only focus on identified microbial species, we may overlook unknown species that also play important roles in the DNRA process. In addition, this method may also bias the understanding of microbial dynamics. Microbial dynamics studies the growth, reproduction, and metabolic processes of microbial populations, as well as their interactions with the environment. In the DNRA community, there may be complex interactions and dependency relationships among various microorganisms, which may affect the functionality and stability of the entire community. If unidentified species are ignored, we may not be able to fully understand these interactions and dependencies, and thus cannot accurately reveal the microbial dynamics mechanism of the DNRA process. Therefore, to gain a more comprehensive and in-depth understanding of the DNRA process and the microbial dynamics involved, we will adopt a wider range of methods in future research, including identifying and analyzing unknown species in the DNRA community. This can be achieved by combining multiple technological approaches, such as metagenomics and metabolomics, to more comprehensively reveal the structure and function of DNRA communities. At the same time, attention should also be paid to avoiding excessive interpretation and bias toward existing data and maintaining the objectivity and rigor of scientific research.

Although previous studies have shown that the DNRA process is mainly driven by fermentative bacteria, the dominant bacteria in our study were respiratory bacteria. Moreover, the effect of sediment erosion and the degree of erosion caused by cascade dams in different construction periods on sediment types also affect microbial community structure (Chen et al., 2019). Research on the Three Gorges Dam has confirmed that serious coarsening of the sediment downstream of the dam changes the grain characteristics of the sediment, resulting in the loss of nutrients (Lai et al., 2017). Similarly, by comparing the locations of different cascade dams, we found that the OC content in the sediments of the Lancang River was different among the different reservoirs. Different DNRA bacteria have different physiological characteristics in the substrate (NO_3_^−^-N and OC), temperature, pH, and nutrient preference (Domangue and Mortazavi, 2018; Wang et al., 2020b; Hernandez-del Amo and Baneras, 2021), which indicates that different DNRA bacteria have genus-specific habitats and ecological niche. Therefore, the severe coarsening distribution of sediments caused by cascade damming inevitably affects the microbial ecological niche and the characteristics of the DNRA bacterial community. In addition, the changes in dominant groups of DNRA bacteria at different water depth sampling sites will show the influence of reservoir engineering factors on certain bacterial communities, which will be more important for determining the key or indicator drivers of dam engineering factors.

Although α-diversity did not significantly affect the DNRA potential rate (Supplementary Table S8), *Anaeromyxobacter*, which had the highest abundance at the genus level, was significantly correlated with the potential rate. This is because, in a specific environment, DNRA may select a few aggregation communities with strong adaptability and low α-diversity. In addition, our results showed that the abundance of DNRA in sandy sediments was higher than that in argillaceous sediments (depending on the sampling points upstream and downstream of the reservoir). Sediment permeability may play an important role in determining the survival rate of DNRA bacterial species. This is not only because of the high permeability of sandy sediments but also because the oxidation environment on the surface promotes the growth of DNRA bacteria. This result supports the important role of sediment type in shaping the diversity of DNRA bacteria in the Lancang River.

### 4.3 The ecological patterns and networks of DNRA bacterial community composition

There have been many reports on the biogeography of N cycle functional bacteria, but the research has mainly focused on denitrification and anammox bacterial communities and few on the biogeography of DNRA bacteria (Li et al., 2018; Mao et al., 2019; Chen et al., 2020b). In previous studies, temperature, redox potential, pH, and C/N ratio are the most important factors for the formation of the DNRA bacterial community, which have been discussed (Pandey et al., 2020; Hernandez-del Amo and Baneras, 2021). However, the influence of geospatial factors on the biogeographical patterns of the DNRA community remains unclear. We found strong evidence for the environmental distance attenuation relationship of the DNRA bacterial community in the cascade reach of the Lancang River, indicating that homogeneity selection is a key factor affecting the accumulation of the anammox bacterial community. The slope of the distance-decay curve of the DNRA bacterial community was similar to that of the total bacteria reported previously (Wang et al., 2017; Meyer et al., 2018). This was less than the distance attenuation slope of other functional bacteria including ammonia-oxidizing bacteria (*z* = 0.14), and anammox bacteria (*z* = 0.35) (Chen et al., 2020b). Previous studies on anammox bacteria have found that it has high auto-aggregation and adhesion abilities, and the strong filtration ability of sandy sediment is more suitable for the survival of bacteria with good adhesion abilities (Hou et al., 2015; Pandey et al., 2020). However, compared with sandy sediments, previous studies have found that some fine soil particles (clays) can increase the relative abundance of DNRA bacteria by increasing the water-filled pore space (WFPS) and reducing the soil redox potential (Sgouridis et al., 2011; Chen et al., 2015), indicating that DNRA bacteria are more suitable for survival in clay sediments with low redox sites. It is generally believed that selection produces a distance-decay relationship, and dispersal offsets it (Hanson et al., 2012). Therefore, the dispersal ability of DNRA bacteria in the sediment was slightly higher than that of other functional bacteria, resulting in the species turnover rates of the DNRA bacterial community being lower than that of other functional bacterial communities.

Recent studies have confirmed that theories such as physicochemical characteristics (Liu et al., 2018b), riparian effects (Chen et al., 2018), reservoir effects (Lu et al., 2020; Chen et al., 2020a), and neutral processes (Li et al., 2019) can be used to explain the biogeographic patterns of bacterial diversity in rivers with large hydropower dams. Clarifying the possible driving forces and their contribution to the biogeographical pattern of the DNRA bacterial community is essential for better understanding the assembly process of DNRA bacteria. Our results show that environmental distance, rather than spatial geographic distance, has a significant effect on the similarity of the DNRA bacterial community in the sediments of the Lancang River cascade reservoirs. These results confirm that environmental selection rather than diffusion restriction, may significantly affect the bacterial community composition of DNRA in the Lancang River. In an extensive investigation of terrestrial soil ecosystems, DNRA bacteria were not ubiquitous in the soil (Nelson et al., 2016), indicating that the dispersal of these bacteria may also be limited to a certain extent. Therefore, environmental selection plays an important role in the formation of biogeographical patterns in the DNRA bacterial community. In addition, there may be differences in spatial scale, season size, and microbial diversity between our study and other studies, which leads to a weaker distance attenuation pattern of the DNRA bacterial community than that of other functional bacteria.

Moreover, the properties of sediments are the key factors for the formation of the DNRA bacterial community (Pan et al., 2020; Pandey et al., 2020; Wang et al., 2020b). Previous studies have described the DNRA bacteria usually account for a very small part of the total bacteria in the sediments (Wang et al., 2018; Luo et al., 2019). Therefore, the characteristics of the total bacterial community may not fully reflect the DNRA bacteria. A significant relationship was found between environmental and dam engineering factors and DNRA bacteria. However, no significant correlation was observed between geographic spatial factors and the DNRA bacterial community. Therefore, due to the influence of cascade reservoir construction and watershed characteristics, the geographical spatial factors may not be sufficient to lead to significant variations in the DNRA bacterial community in the cascade reach of the Lancang River (0–750 km), nor to lead to the attenuation of DNRA bacterial distance in the watershed. Compared with the study of the entire Yangtze River Basin (Chen et al., 2020b), environmental selection plays a more important role in the formation of the pattern of DNRA bacteria.

Co-occurrence patterns have been successfully applied to the analysis of microbial species in a variety of environments and have great advantages in revealing specific but less obvious relationships in complex community datasets (Li et al., 2015a; Zou et al., 2017; Han et al., 2020; Li et al., 2021; Zhang et al., 2021a). In this study, the summer and winter networks only had one independent module. Some studies interpret modules as niches (Zhang et al., 2018b, 2021a); if the number of modules is greater, niche differentiation is stronger. A co-occurrence correlation analysis showed that the number of positive correlations among DNRA bacterial communities was more than that of negative correlations, which may indicate that the DNRA bacterial community in Lancang cascade reservoirs has matured, the response trend of different DNRA bacterial ASVs to environmental parameters was the same, and there was less competition among microorganisms in this habitat (Mikhailov et al., 2019). Therefore, DNRA bacteria from different cascade reservoirs can effectively share similar niches. The small difference in niche preference among the members of the DNRA bacterial community may not be enough to change the response of the DNRA bacterial community to spatial changes in environmental factors. In addition, our study also found that although some key ASVs are not the most abundant groups in the network, they occupy an important position in the network, and may play a key role in mediating network interaction and maintaining DNRA activity in sediments and overall nitrate dissimilatory reduction metabolism function (Banerjee et al., 2016; Pandey et al., 2020; Zhang et al., 2021a).

## 5 Conclusion

In conclusion, our study explored the spatio-temporal characteristics, geographical patterns, symbiotic network, and environmental driving factors of DNRA bacteria in the cascade reservoirs of the Lancang River, and improved our understanding of the biogeochemical cycles related to nitrogen in large-scale hydropower development rivers. The potential rate and composition of DNRA bacteria along the gradient of the cascade reservoirs showed significant spatial heterogeneity, but the seasonal difference was not obvious. The distribution of sediment fragmentation caused by cascade damming affected the niche and community characteristics of DNRA bacteria, whereas *Anaeromyxobacter*, the most abundant bacteria in horizontal abundance, showed a significant correlation with the potential rate. The Pearson correlation analysis and RDA showed that the potential rate of DNRA was determined by T, NH_4_^+^-N in the sediments, water depth, and reservoir age. Simultaneously, the similarities of the DNRA bacterial community in the surface sediments of cascade reservoirs show a certain distance attenuation relationship and the deterministic environmental selection process plays a crucial role in the formation of the DNRA bacterial community. The co-occurrence correlation analysis showed that the number of positive correlations among DNRA bacterial communities was greater than that of negative correlations, and DNRA bacteria from different cascade reservoirs could effectively share similar niches. Our observations are of great significance for the future study of the geographical pattern of N cycling microorganisms in rivers across latitudes, and further research is needed to confirm the universality of the DNRA community environmental selection process, to provide a reference for other cascade development rivers.

## Data availability statement

The original contributions presented in the study are included in the article/Supplementary material, further inquiries can be directed to the corresponding author.

## Author contributions

BY: Writing – review & editing, Writing – original draft, Supervision, Project administration, Methodology, Investigation, Funding acquisition, Formal analysis, Data curation, Conceptualization. MG: Writing – review & editing, Resources, Investigation. XZ: Writing – review & editing, Resources, Project administration. ML: Writing – original draft, Methodology, Investigation, Data curation. SX: Writing – review & editing, Visualization, Validation, Supervision, Methodology, Conceptualization.

## Footnotes

^1^ https://gephi.org
