# Supplementary material for: Spatiotemporal patterns and co-occurrence patterns of dissimilatory nitrate reduction to ammonium community in sediments of the Lancang River cascade reservoirs
Source: Front Microbiol. 2024 Jun 19;15:1411753. doi: 10.3389/fmicb.2024.1411753 (PMC11219630; doi:10.3389/fmicb.2024.1411753)
Supplement: Supplementary file 1 [file Data_Sheet_1.docx]

Supplementary Material

# Supplementary Tables

**Supplementary Table S1. Main characteristics of Cascade Hydropower Stations in Yunnan Section of Lancang River**

| **Main indicators** | **Miaowei**  **(M)** | **Gongguoqiao (GGQ)** | **Xiaowan**  **(XW)** | **Manwan**  **(MW)** | **Dachaoshan**  **(DCS)** | **Nuozhadu**  **(NZD)** | **Jinghong**  **(JH)** |
| --- | --- | --- | --- | --- | --- | --- | --- |
| **Catchment Area / km^2^** | 93900 | 97200 | 113300 | 114500 | 121000 | 144700 | 149100 |
| **Average Water Flow / (m^3^ /s)** | 960 | 985 | 1220 | 1230 | 1340 | 1750 | 1840 |
| **Dam Height / m** | 131 | 130 | 300 | 126 | 110 | 254 | 118 |
| **Reservoir Surface Area / km^2^** | 14.26 | 16.72 | 189.1 | 23.6 | 26.25 | 320 | 32.81 |
| **Backwater Length / km** | 60 | 52 | 178 | 70 | 80 | 210 | 105 |
| **Operating Water Level / m** | 1408 | 1319 | 1236 | 994 | 895 | 807 | 602 |
| **Effective Storage Capacity / ( ×10^8^m^3^)** | 165 | 120 | 9900 | 257 | 367 | 12300 | 249 |
| **Total Reservoir Volume / ( ×10^8^m^3^)** | 772 | 510 | 14560 | 920 | 933 | 22400 | 1233 |
| **Hydrological Residence Time / y** | 0.02 | 0.01 | 2.36 | 0.78 | 0.3 | 1.87 | 0.4 |
| **Installed Capacity / MW** | 1400 | 750 | 4200 | 1500 | 1350 | 5850 | 1750 |
| **Regulation Pattern** | Adjusted Weekly | Adjusted Daily | Not Yearly Adjusted | Not Seasonally Adjusted | Not Seasonally Adjusted | Not Yearly Adjusted | Not Seasonally Adjusted |
| **Impoundment time** | 2016 | 2011 | 2008 | 1993 | 2001 | 2011 | 2008 |

**Supplementary Table S2.** **Latitude, longitude and altitude of sediment sampling sites in Lancang River**

| **Abbreviations** | **Longitude (°E)** | **Latitude (°N)** | **Distance to the frontier (km)** | **Elevation (m)** |
| --- | --- | --- | --- | --- |
| **MW01** | 99.14 | 26.2 | 794 | 1382 |
| **MW02** | 99.16 | 25.85 | 754 | 1311 |
| **GGQ01** | 99.23 | 25.76 | 732 | 1296 |
| **GGQ02** | 99.33 | 25.59 | 713 | 1259 |
| **XW01** | 99.73 | 24.97 | 605 | 1105 |
| **XW02** | 100.13 | 24.74 | 557 | 1046 |
| **HHJ01** | 100.09 | 24.71 | 548 | 1045 |
| **MW01** | 100.1 | 24.67 | 539 | 1007 |
| **MW02** | 100.4 | 24.63 | 489 | 994 |
| **DCS01** | 100.49 | 24.54 | 476 | 898 |
| **DCS02** | 100.37 | 24.04 | 396 | 857 |
| **NZD01** | 100.05 | 23.2 | 273 | 692 |
| **NZD02** | 100.4 | 22.66 | 191 | 622 |
| **JH01** | 100.58 | 22.5 | 161 | 585 |
| **JH02** | 100.72 | 22.12 | 99 | 564 |

**Table S3 Physicochemical variables of sediment samples in Lancang River**

|  | **T**  **(℃)** | **pH** | **EC**  **(μm s^-1^)** | **Moisture (%)** | **TOC**  **(%)** | **TP**  **(mg/kg)** | **TN**  **(mg/kg)** | **NH_4_^+^-N (mg/kg)** | **NO_3_^-^-N**  **(mg/kg)** | **NO_2_^-^-N**  **(mg/kg)** |
| --- | --- | --- | --- | --- | --- | --- | --- | --- | --- | --- |
| **SM01** | 18.03 ± 0.16 | 8.07 ± 0.09 | 42.75 ± 9.48 | 21.50 ± 2.81 | 0.61 ± 0.05 | 553.87 ± 18.52 | 791.55 ± 80.21 | 20.07 ± 2.37 | 2.53± 0.71 | 0.07 ± 0.01 |
| **SM02** | 18.12 ± 0.23 | 8.14 ± 0.11 | 49.31 ± 13.27 | 19.13 ± 4.28 | 0.93 ± 0.14 | 613.04 ± 23.57 | 838.75 ± 107.57 | 17.33± 3.06 | 1.79 ± 1.03 | 0.09 ± 0.02 |
| **SGGQ01** | 18.47 ± 0.15 | 8.34 ± 0.06 | 60.65 ± 20.9 | 22.83 ± 3.77 | 1.05 ± 0.17 | 550.43 ± 50.66 | 745.23 ± 125.96 | 16.00 ± 0.88 | 1.90 ± 0.33 | 0.05 ± 0.02 |
| **SGGQ02** | 18.60 ± 0.10 | 8.41 ± 0.04 | 50.06 ± 11.21 | 22.73 ± 4.29 | 0.49 ± 0.04 | 631.87 ± 35.54 | 949.17 ± 60.00 | 16.12 ± 1.83 | 2.75 ± 0.24 | 0.09 ± 0.02 |
| **SXW01** | 19.43 ± 0.15 | 7.86 ± 0.10 | 32.05 ± 7.63 | 23.23 ± 5.42 | 0.26 ± 0.09 | 666.08 ± 61.23 | 1297.17 ± 115.51 | 13.71 ± 1.68 | 3.43 ± 0.53 | 0.10 ± 0.03 |
| **SXW02** | 19.30 ± 0.26 | 8.15 ± 0.08 | 27.54 ± 8.31 | 28.27 ± 4.99 | 0.60 ± 0.06 | 894.51 ± 38.05 | 1656.31 ± 147.07 | 23.99 ± 4.15 | 4.04 ± 0.45 | 0.09 ± 0.02 |
| **SHHJ01** | 18.97 ± 0.13 | 7.23 ± 0.09 | 43.1 ± 14.83 | 20.00 ± 4.48 | 0.48 ± 0.06 | 799.17 ± 36.21 | 960.71 ± 191.21 | 15.46 ± 2.23 | 2.46 ± 0.62 | 0.07 ± 0.01 |
| **SMW01** | 19.47 ± 0.15 | 8.22 ± 0.13 | 39.22 ± 13.87 | 24.47 ± 5.47 | 0.18 ± 0.10 | 613.73 ± 26.80 | 732.41 ± 91.94 | 32.69 ± 3.96 | 4.31 ± 0.64 | 0.09 ± 0.03 |
| **SMW02** | 19.53 ± 0.21 | 7.74 ± 0.06 | 36.21 ± 10.22 | 23.50 ± 4.81 | 1.01 ± 0.12 | 806.61 ± 20.99 | 1888.54 ± 159.54 | 32.97 ± 4.65 | 4.54 ± 0.63 | 0.10 ± 0.03 |
| **SDCS01** | 20.37 ± 0.16 | 7.79 ± 0.14 | 29.73 ± 11.15 | 18.17 ± 3.48 | 0.39 ± 0.06 | 560.79 ± 40.83 | 1030.21 ± 124.96 | 21.67 ± 1.93 | 2.57 ± 0.35 | 0.06 ± 0.03 |
| **SDCS02** | 20.43 ± 0.45 | 7.81 ± 0.08 | 21.83 ± 9.42 | 26.03 ± 3.69 | 0.77 ± 0.03 | 961.08 ± 19.66 | 1623.05 ± 100.56 | 17.55 ± 2.27 | 2.52 ± 0.53 | 0.09 ± 0.04 |
| **SNZD01** | 16.47 ± 0.21 | 7.39 ± 0.05 | 28.8 ± 12.31 | 19.67 ± 1.99 | 0.39 ± 0.02 | 474.07 ± 27.65 | 978.98 ± 107.91 | 20.21 ± 1.30 | 2.44 ± 0.66 | 0.06 ± 0.02 |
| **SNZD02** | 16.54 ± 0.25 | 8.02 ± 0.07 | 37.75 ± 13.08 | 25.87 ± 3.27 | 0.82 ± 0.06 | 632.80 ± 39.85 | 1052.35 ± 103.28 | 16.04 ± 2.14 | 2.81 ± 0.44 | 0.08 ± 0.02 |
| **SJH01** | 27.80 ± 0.44 | 6.99 ± 0.11 | 52.41 ± 15.87 | 23.13 ± 3.76 | 0.52 ± 0.04 | 575.20 ± 34.46 | 1030.74 ± 84.88 | 14.83 ± 1.95 | 2.24 ± 0.31 | 0.08 ± 0.01 |
| **SJH02** | 27.58 ± 0.13 | 7.53 ± 0.05 | 33.18 ± 7.91 | 26.10 ± 3.97 | 0.92 ± 0.03 | 764.63 ± 51.20 | 1763.30 ± 183.26 | 20.33 ± 2.99 | 2.87 ± 0.52 | 0.11 ± 0.02 |
| **WM01** | 8.83±0.12 | 8.12 ± 0.04 | 45.45 ± 12.16 | 31.03 ± 3.07 | 0.63 ± 0.06 | 632.93 ± 31.50 | 865.23 ± 50.76 | 17.82 ± 1.07 | 2.18± 0.27 | 0.05v± 0.02 |
| **WM02** | 9.07±0.06 | 8.03 ± 0.04 | 40.88 ± 14.31 | 31.00 ± 4.02 | 0.89 ± 0.08 | 650.27 ± 90.26 | 1063.82 ± 114.72 | 15.32 ± 2.71 | 2.45 ± 0.70 | 0.06 ± 0.02 |
| **WGGQ01** | 11.17 ± 0.12 | 8.33 ± 0.03 | 35.13 ± 15.47 | 23.61 ± 1.07 | 0.44 ± 0.18 | 485.14 ± 45.34 | 785.32 ± 52.38 | 17.46 ± 0.83 | 2.64 ± 0.52 | 0.07 ± 0.01 |
| **WGGQ02** | 12.30 ± 0.20 | 8.40 ± 0.05 | 48.21 ± 18.17 | 26.47 ± 3.32 | 0.85 ± 0.13 | 710.95 ± 18.97 | 1012.40 ± 95.20 | 17.42 ± 1.04 | 2.89 ± 0.61 | 0.11 ± 0.03 |
| **WXW01** | 13.43 ± 0.15 | 7.84 ± 0.07 | 55.74 ± 16.71 | 20.14 ± 0.74 | 0.47 ± 0.01 | 522.51 ± 18.97 | 1209.67 ± 30.97 | 14.26 ± 4.55 | 3.54 ± 0.50 | 0.12 ± 0.02 |
| **WXW02** | 13.30 ± 0.26 | 8.16 ± 0.08 | 27.91 ± 7.38 | 34.90 ± 1.52 | 0.86 ± 0.09 | 854.67 ± 39.87 | 1586.97 ± 52.06 | 25.54 ± 9.01 | 4.64 ± 1.16 | 0.11 ± 0.04 |
| **WHHJ01** | 13.97 ± 0.13 | 7.39 ± 0.05 | 38.76 ± 8.38 | 34.57 ± 2.25 | 0.71 ± 0.06 | 731.87 ± 42.36 | 917.85 ± 74.62 | 15.58 ± 1.76 | 2.86 ± 1.35 | 0.07 ± 0.03 |
| **WMW01** | 14.27 ± 0.21 | 8.23 ± 0.06 | 40.51 ± 10.33 | 29.53 ± 3.20 | 0.28 ± 0.08 | 590.31 ± 47.72 | 647.77 ± 55.60 | 15.75 ± 3.48 | 4.18 ± 0.89 | 0.09 ± 0.02 |
| **WMW02** | 14.53 ± 0.13 | 7.78 ± 0.07 | 34.14 ± 10.82 | 33.63 ± 2.48 | 1.06 ± 0.17 | 773.28 ± 39.01 | 1642.14 ± 133.25 | 24.85 ± 3.86 | 4.77 ± 0.75 | 0.11 ± 0.03 |
| **WDCS01** | 15.50± 0.30 | 7.71 ± 0.10 | 29.57 ± 7.03 | 27.67 ± 4.61 | 0.69 ± 0.07 | 527.60 ± 27.43 | 1053.27 ± 75.40 | 12.70 ± 0.95 | 2.51 ± 0.62 | 0.11 ± 0.02 |
| **WDCS02** | 15.37 ± 0.41 | 7.77 ± 0.04 | 25.73 ± 7.67 | 27.07 ± 3.31 | 1.07 ± 0.05 | 927.76 ± 40.99 | 1485.68 ± 33.74 | 19.88 ± 2.42 | 4.02 ± 1.03 | 0.09 ± 0.03 |
| **WNZD01** | 18.93 ± 0.35 | 7.38± 0.06 | 33.58 ± 9.21 | 19.59 ± 3.76 | 0.37 ± 0.05 | 438.99 ± 24.97 | 794.30 ± 30.92 | 20.35 ± 2.01 | 3.15 ± 0.56 | 0.11± 0.03 |
| **WNZD02** | 20.70 ± 0.36 | 8.09± 0.09 | 39.09 ±11.34 | 21.78 ± 7.28 | 0.85 ± 0.05 | 599.47 ± 19.19 | 1031.48 ± 73.30 | 24.08 ± 1.67 | 2.53 ± 0.68 | 0.08 ± 0.04 |
| **WJH01** | 22.03± 0.31 | 7.11± 0.12 | 38.52 ± 9.07 | 32.01± 3.58 | 0.78 ± 0.10 | 554.67± 33.92 | 838.59 ± 41.58 | 30.93 ± 2.53 | 2.83 ± 0.28 | 0.09 ± 0.02 |
| **WJH02** | 22.90± 0.36 | 7.56 ± 0.05 | 36.89 ± 12.17 | 30.13 ± 1.96 | 1.07 ± 0.14 | 732.29 ± 36.23 | 1663.82 ± 245.88 | 18.65 ± 3.55 | 2.68 ± 0.33 | 0.10 ± 0.04 |

**Supplementary Table S4. Statistics of sequencing results. Generated by QIIME2**

| **SampleID** | **Input** | **Merged** | **Nonchimeric** | **Nonsingleton** |
| --- | --- | --- | --- | --- |
| **SM01** | 135030 | 133026 | 130830 | 118752 |
| **SM02** | 130774 | 129068 | 127002 | 116468 |
| **SGGQ01** | 128626 | 127372 | 125356 | 118555 |
| **SGGQ02** | 139409 | 137491 | 135156 | 124287 |
| **SXW01** | 141822 | 139833 | 137513 | 130915 |
| **SXW02** | 134821 | 132934 | 130693 | 124120 |
| **SHHJ01** | 115034 | 113060 | 111066 | 106715 |
| **SMW01** | 134523 | 133151 | 131130 | 120994 |
| **SMW02** | 135798 | 134404 | 132119 | 122381 |
| **SDCS01** | 140382 | 138736 | 136584 | 126428 |
| **SDCS02** | 115940 | 114406 | 112676 | 104053 |
| **SNZD01** | 93454 | 90821 | 89432 | 84376 |
| **SNZD02** | 83795 | 80961 | 79511 | 74199 |
| **SJH01** | 93002 | 91925 | 90357 | 83223 |
| **SJH02** | 94842 | 93687 | 92256 | 82450 |
| **WM01** | 107658 | 106250 | 104676 | 97895 |
| **WM02** | 123889 | 122108 | 120301 | 110930 |
| **WGGQ01** | 142849 | 140810 | 138832 | 125483 |
| **WGGQ02** | 102718 | 101115 | 99620 | 94525 |
| **WXW01** | 110709 | 109402 | 107850 | 102774 |
| **WXW02** | 142691 | 140437 | 137713 | 128362 |
| **WHHJ01** | 118812 | 115915 | 113898 | 108526 |
| **WMW01** | 141860 | 140214 | 137830 | 125741 |
| **WMW02** | 137516 | 135455 | 133111 | 115042 |
| **WDCS01** | 131526 | 129643 | 127345 | 111619 |
| **WDCS02** | 110113 | 108503 | 106395 | 97187 |
| **WNZD01** | 118629 | 113894 | 111494 | 104540 |
| **WNZD02** | 139658 | 136477 | 133952 | 125238 |
| **WJH01** | 144023 | 141834 | 139664 | 129190 |

**Supplementary Table S5. Bacterial diversity of the reservoir sediments samples based on the Miseq sequencing of 16S rRNA genes.**

| **Sample** | **Chao1** | **Goods**  **coverage** | **Observed**  **species** | **Pielou-e** | **Shannon** | **Simpson** |
| --- | --- | --- | --- | --- | --- | --- |
| **SM01** | 1399.32 | 0.994 | 1368.8 | 0.937 | 9.757 | 0.998 |
| **WM01** | 1648.74 | 0.990 | 1587.3 | 0.938 | 9.976 | 0.998 |
| **WM02** | 1723.9 | 0.989 | 1654.5 | 0.927 | 9.912 | 0.998 |
| **SGGQ01** | 1591.75 | 0.989 | 1520.1 | 0.914 | 9.661 | 0.998 |
| **SGGQ02** | 1668.21 | 0.988 | 1585.7 | 0.909 | 9.662 | 0.997 |
| **WGGQ01** | 1356.48 | 0.990 | 1288.5 | 0.791 | 8.167 | 0.963 |
| **WGGQ02** | 1326.99 | 0.993 | 1288.3 | 0.882 | 9.110 | 0.993 |
| **SXW01** | 1390.9 | 0.992 | 1337.4 | 0.898 | 9.324 | 0.995 |
| **SXW02** | 1255.49 | 0.994 | 1223.9 | 0.886 | 9.090 | 0.993 |
| **SHHJ01** | 1319.5 | 0.994 | 1291.4 | 0.915 | 9.457 | 0.997 |
| **WXW01** | 1279.67 | 0.995 | 1256.8 | 0.933 | 9.602 | 0.998 |
| **WXW02** | 1230.88 | 0.995 | 1208 | 0.916 | 9.377 | 0.997 |
| **WHHJ01** | 1192.22 | 0.997 | 1185.3 | 0.941 | 9.609 | 0.998 |
| **SMW01** | 1327.21 | 0.991 | 1263.1 | 0.907 | 9.340 | 0.997 |
| **SMW02** | 1315.69 | 0.992 | 1257 | 0.913 | 9.403 | 0.997 |
| **WMW01** | 1428.5 | 0.992 | 1380 | 0.929 | 9.692 | 0.998 |
| **WMW02** | 1543.61 | 0.991 | 1488 | 0.937 | 9.870 | 0.998 |
| **SDCS01** | 1188.64 | 0.993 | 1139.8 | 0.880 | 8.941 | 0.993 |
| **SDCS02** | 1402.18 | 0.992 | 1355.4 | 0.932 | 9.700 | 0.998 |
| **WDCS01** | 1367.36 | 0.995 | 1341.2 | 0.943 | 9.800 | 0.998 |
| **WDCS02** | 1512.2 | 0.992 | 1462.1 | 0.944 | 9.922 | 0.999 |
| **SNZD01** | 1443.96 | 0.992 | 1396.4 | 0.939 | 9.810 | 0.998 |
| **SNZD02** | 1162.57 | 0.992 | 1105.6 | 0.862 | 8.712 | 0.990 |
| **WNZD01** | 1428.88 | 0.994 | 1395.5 | 0.929 | 9.702 | 0.998 |
| **WNZD02** | 1175.04 | 0.996 | 1154.3 | 0.927 | 9.435 | 0.997 |
| **SJH01** | 1301.4 | 0.993 | 1250.1 | 0.915 | 9.416 | 0.997 |
| **SJH02** | 1465.03 | 0.991 | 1408.6 | 0.932 | 9.744 | 0.998 |
| **WJH01** | 1729.13 | 0.988 | 1642.7 | 0.926 | 9.891 | 0.998 |
| **WJH02** | 1422.07 | 0.994 | 1389.4 | 0.941 | 9.826 | 0.998 |

**Supplementary Table S6. Two-way crossed analysis of similarity (ANOSIM) of DNRA bacterial community composition based on Bray-Curtis dissimilarity across seasons and reservoirs (999 permutations).**

| **Group 1** | **Group 2** | | | **R** | **P** | |  |
| --- | --- | --- | --- | --- | --- | --- | --- |
| ***Season*** |  | | |  |  | |  |
| Global ANOSIM |  | | | -0.022 | 0.598 | |  |
| S | W | | | -0.022 | 0.602 | |  |
| ***Reservoirs*** | |  |  | | |  | |
| Global ANOSIM |  | | | 0.738 | 0.001 | |  |
| M | GGQ | | | 0.375 | 0.024 | |  |
| M | XW | | | 0.988 | 0.005 | |  |
| M | MW | | | 1.000 | 0.033 | |  |
| M | DCS | | | 1.000 | 0.038 | |  |
| M | NZD | | | 1.000 | 0.027 | |  |
| M | JH | | | 0.917 | 0.035 | |  |
| GGQ | XW | | | 0.992 | 0.002 | |  |
| GGQ | MW | | | 0.979 | 0.033 | |  |
| GGQ | DCS | | | 0.990 | 0.034 | |  |
| GGQ | NZD | | | 1.000 | 0.027 | |  |
| GGQ | JH | | | 0.906 | 0.024 | |  |
| XW | MW | | | 0.873 | 0.005 | |  |
| XW | DCS | | | 0.821 | 0.004 | |  |
| XW | NZD | | | 0.579 | 0.005 | |  |
| XW | JH | | | 0.619 | 0.006 | |  |
| MW | DCS | | | 0.854 | 0.026 | |  |
| MW | NZD | | | 0.969 | 0.034 | |  |
| MW | JH | | | 0.615 | 0.033 | |  |
| DCS | NZD | | | 0.833 | 0.025 | |  |
| DCS | JH | | | 0.417 | 0.030 | |  |
| NZD | JH | | | 0.052 | 0.434 | |  |

**Supplementary Table S7. Two-way crossed analysis of similarity (ANOSIM) of bacterial community composition based on Bray-Curtis dissimilarity across seasons and reservoirs (999 permutations).**

| **season** | **topo** | **empirical network** | **random network avg** | **random network sd** |
| --- | --- | --- | --- | --- |
| **Summer** | Average nearest neighbor degree | 301.3867 | 266.7744 | 0.0279 |
|  | Average path length | 2.097 | 1.8705 | 0 |
|  | Betweenness centrality | 12896137.98 | 796309.3526 | 109496.5395 |
|  | Closeness centrality | 135.8659 | 15.4103 | 1.9648 |
|  | Degree assortativity | 0.4596 | -0.0009 | 0.0016 |
|  | Degree centralization | 649252 | 109255.4 | 13708.7297 |
|  | Density | 0.1295 | 0.1295 | 0 |
|  | Cluster number | 1 | 1 | 0 |
|  | Diameter | 2.047 | 2 | 0 |
|  | Transitivity | 0.5108 | 0.1296 | 0.0001 |
|  | Number vertice | 2054 | 2054 | 0 |
|  | Number edge | 273088 | 273088 | 0 |
|  | Modularity | 0.5547 | 0.0482 | 0.0009 |
| **Winter** | Average nearest neighbor degree | 390.8402 | 331.229 | 0.0331 |
|  | Average path length | 2.0628 | 1.8599 | 0 |
|  | Betweenness centrality | 19378318.23 | 905546.8815 | 62277.2746 |
|  | Closeness centrality | 206.9409 | 16.7193 | 1.2542 |
|  | Degree assortativity | 0.5678 | -0.0008 | 0.001 |
|  | Degree centralization | 1138986 | 134806 | 9966.1694 |
|  | Density | 0.1401 | 0.1401 | 0 |
|  | Cluster number | 1 | 1 | 0 |
|  | Diameter | 2.0451 | 2 | 0 |
|  | Transitivity | 0.5957 | 0.14 | 0.0001 |
|  | Number vertice | 2360 | 2360 | 0 |
|  | Number edge | 389847 | 389847 | 0 |
|  | Modularity | 0.5673 | 0.0428 | 0.0006 |

**Table S8 Pearson correlation analysis of DNRA bacterial communities with environmental factors**

|  | **abundance** | **rate** | ***Anaeromyxobacter*** | ***Polyangium*** | ***Archangium*** | ***Geothrix*** | ***Lacunisphaera*** | ***Lacunisphaera*** | **Chao1** | **Goods** | **Observed** | **Pielou’s** | **Shannon** | **Simpson** |
| --- | --- | --- | --- | --- | --- | --- | --- | --- | --- | --- | --- | --- | --- | --- |
| **T** | **-0.423*** | 0.296 | 0.075 | -0.102 | -0.300 | **0.425*** | **0.475*** | **0.476*** | -0.159 | 0.131 | -0.190 | **-0.433*** | **-0.373*** | **-0.404*** |
| **Altitude** | **0.433*** | 0.027 | 0.032 | 0.064 | **0.461*** | **-0.617**** | -0.267 | -0.267 | 0.113 | -0.083 | 0.161 | 0.329 | 0.306 | 0.224 |
| **WD** | -0.139 | **-0.429*** | **-0.412*** | -0.113 | **-0.408*** | 0.138 | -0.169 | -0.169 | -0.328 | 0.322 | -0.321 | -0.296 | -0.335 | -0.201 |
| **RA** | 0.038 | **0.436*** | **0.504*** | 0.030 | -0.222 | **0.384*** | -0.071 | -0.071 | 0.139 | -0.149 | 0.064 | 0.029 | 0.038 | 0.155 |
| **WRT** | -0.064 | -0.164 | -0.343 | 0.013 | **-0.676**** | **0.369*** | **-0.529*** | -0.529 | -0.367 | 0.375 | -0.379 | -0.355 | -0.381 | -0.224 |
| **Sand** | **0.445*** | 0.262 | 0.181 | 0.156 | -0.139 | -0.125 | -0.058 | -0.058 | -0.103 | 0.104 | -0.099 | 0.071 | 0.017 | 0.142 |
| **Clay** | -0.131 | 0.101 | -0.158 | -0.314 | **-0.417*** | 0.284 | 0.317 | 0.317 | 0.131 | -0.129 | 0.140 | 0.226 | 0.198 | 0.175 |
| **Silt** | -0.355 | -0.279 | -0.098 | -0.021 | 0.244 | 0.003 | -0.070 | -0.070 | 0.050 | -0.052 | 0.041 | -0.148 | -0.088 | -0.193 |
| **pH** | **0.395*** | 0.176 | 0.118 | 0.141 | **0.442*** | **-0.485*** | -0.360 | -0.360 | -0.066 | 0.087 | -0.053 | 0.065 | 0.029 | 0.035 |
| **Moisture** | -0.177 | 0.053 | 0.117 | -0.100 | -0.022 | 0.312 | -0.086 | -0.086 | -0.180 | 0.191 | -0.206 | -0.099 | -0.149 | 0.053 |
| **OC** | **-0.394*** | 0.101 | -0.015 | -0.034 | 0.053 | 0.183 | -0.009 | -0.009 | 0.093 | -0.083 | 0.067 | -0.005 | 0.016 | 0.033 |
| **TOC** | -0.332 | -0.192 | -0.024 | -0.170 | -0.329 | 0.262 | -0.099 | -0.099 | -0.302 | 0.287 | -0.333 | **-0.417*** | **-0.419*** | -0.199 |
| **TP** | -0.144 | -0.118 | 0.033 | -0.134 | -0.033 | 0.042 | -0.129 | -0.129 | -0.043 | 0.054 | -0.050 | -0.083 | -0.083 | 0.014 |
| **TN** | -0.272 | -0.098 | 0.107 | -0.101 | -0.142 | 0.000 | -0.055 | -0.055 | -0.013 | 0.000 | -0.048 | -0.231 | -0.187 | -0.120 |
| **C/N** | 0.040 | 0.000 | -0.218 | -0.081 | -0.257 | 0.318 | -0.072 | -0.072 | -0.295 | 0.300 | -0.286 | -0.073 | -0.151 | 0.031 |
| **NH_4_^+^-N** | -0.105 | **0.602**** | 0.321 | 0.110 | -0.166 | 0.360 | -0.218 | -0.218 | -0.139 | 0.139 | -0.198 | -0.177 | -0.199 | 0.016 |
| **NO_3_^-^-N** | 0.089 | 0.286 | 0.244 | 0.030 | -0.250 | -0.061 | -0.276 | -0.276 | -0.156 | 0.151 | -0.201 | -0.191 | -0.216 | -0.020 |
| **NO_2_^-^-N** | -0.307 | 0.019 | 0.199 | 0.147 | -0.177 | 0.046 | -0.078 | -0.078 | 0.007 | -0.043 | -0.036 | -0.339 | -0.266 | -0.287 |

WD: Water depth; RA: Reservoir age

* represents P < 0.05; ** represents P < 0.01.

**Table S9 Studies on DNRA and methods followed and major findings in different ecosystems.**

| **Research region/Country** | **Ecosystem types** | **Methodology** | **Findings** | **Reference** |
| --- | --- | --- | --- | --- |
| China | Coastal wetland | ^15^N tracing technique | DNRA could be controlled by organic carbon sources, especially organic acids. Without exogenous C showed low soil DNRA rates, presumably because of the low C/NO_3_^−^ ratio as well as energy availability. | Liu et al. (2016) |
| Australia | River estuary sediment | ^15^N reduction assay | Reduced rates of DNRA under anoxic conditions due to binding of Fe^2+^ with free sulfides and the formation of FeS removing available Fe^2+^ for DNRA. | Robertson et al. (2016) |
| Germany | Freshwater sediment | ^15^N-labeled chemical NO donor SNAP | Initial NO production rate is in the same range as the net ammonia oxidation rate, indicating that NO is transiently the main product of ammonia oxidizers. | Schreiber et al. (2014) |
| Netherlands | Temperate estuary | Nrf gene sequence, Culturable | The study demonstrates high small scale heterogeneity among dissimilatory  nitrate reduction processes in estuarine sediments. | Decleyre et al. (2015) |
| The upper Barataria Bay region | soils and sediments | ^15^N tracing technique | DNRA in wetland soils and benthic sediment is not a major nitrogen transformation in oligohaline regions across the MRDP regardless of wide range of OM% content in these eroding and prograding delta lobes. | Upreti et al. (2022) |
| China | surface sediments | ^15^N isotope incubation | DNRA abundance and rates were significantly correlated with keystone species. Stochastic processes play an important role in shaping DNRA community structure. | Zhao et al. (2022) |
| Spain | highly-saline lake sediments | ^15^N-IPT approach | Anoxia and darkness favored DNRA and anammox processes over denitrification and therefore to restrict N_2_O emissions to the atmosphere. | Valiente et al. (2022) |
| The Curonian Lagoon (Baltic Sea) | Lake water | ^15^N_2_ technique | DNRA was the main nitrate reduction process in the water. Denitrification was also detected in the water, but was predominant in the sediment. These processes were likely occurring in low-oxygen niches related to cyanobacteria. | Broman et al. (2021) |

**References**

Broman, E., Zilius, M., Samuiloviene, A., Vybernaite-Lubiene, I., Politi, T., Klawonn, I. et al. (2021) Active DNRA and denitrification in oxic hypereutrophic waters. *Water Research* **194**: 116954.

Decleyre, H., Heylen, K., Van Colen, C., and Willems, A. (2015) Dissimilatory nitrogen reduction in intertidal sediments of a temperate estuary: small scale heterogeneity and novel nitrate-to-ammonium reducers. *Frontiers in microbiology* **6**: 1124-1124.

Liu, X., Han, J.-G., Ma, Z.w., Wang, Q., and Li, L. (2016) Effect of Carbon source on dissimilatory nitrate reduction to ammonium in Costal Wetland sediments. *Journal of Soil Science and Plant Nutrition* **16**: 337-349.

Robertson, E., Roberts, K., Burdorf, L., Cook, P., and Thamdrup, B. (2016) Dissimilatory nitrate reduction to ammonium coupled to Fe(II) oxidation in sediments of a periodically hypoxic estuary. *Limnology and Oceanography* **61**.

Schreiber, F., Stief, P., Kuypers, M., and de Beer, D. (2014) Nitric oxide turnover in permeable river sediment. *Limnology and oceanography* **59**: 1310-1320.

Upreti, K., Rivera-Monroy, V.H., Maiti, K., Giblin, A.E., and Castañeda-Moya, E. (2022) Dissimilatory nitrate reduction to ammonium (DNRA) is marginal relative to denitrification in emerging-eroding wetlands in a subtropical oligohaline and eutrophic coastal delta. *Science of The Total Environment* **819**: 152942.

Valiente, N., Jirsa, F., Hein, T., Wanek, W., Prommer, J., Bonin, P., and Gómez-Alday, J.J. (2022) The role of coupled DNRA-Anammox during nitrate removal in a highly saline lake. *Science of The Total Environment* **806**: 150726.

Zhao, Y., Yuan, F., Li, S., Zhang, J., Li, Y., Shi, X. et al. (2022) Assembly mechanism and co-occurrence patterns of DNRA microbial communities and imprint of nitrate reduction in the Songhua River sediments of China's largest old industrial base. *Journal of Environmental Management* **322**: 116091.

# Supplementary Figures

**
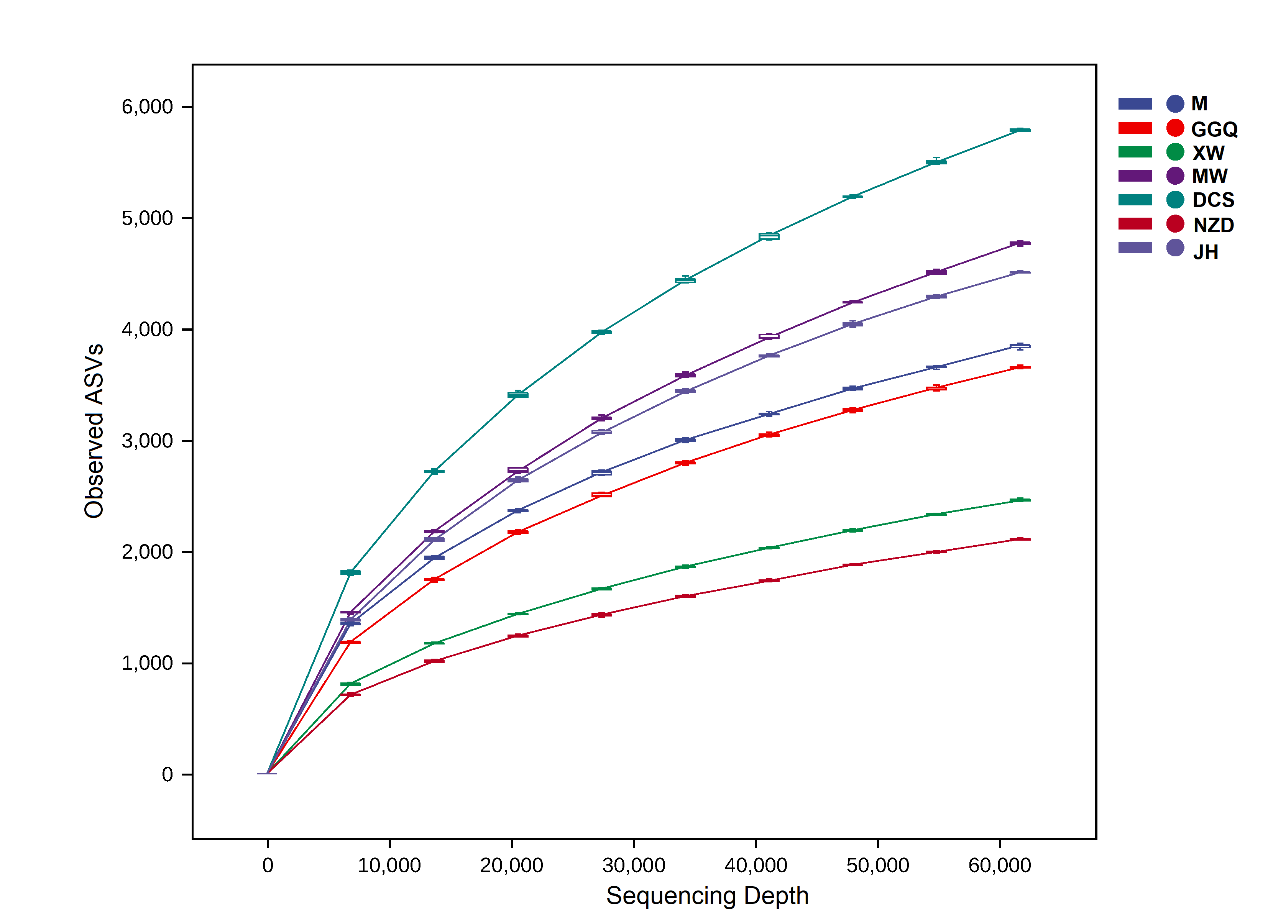
**

**Supplementary Figure S1. Rarefaction curves of different samples of cascade reservoirs.**

**
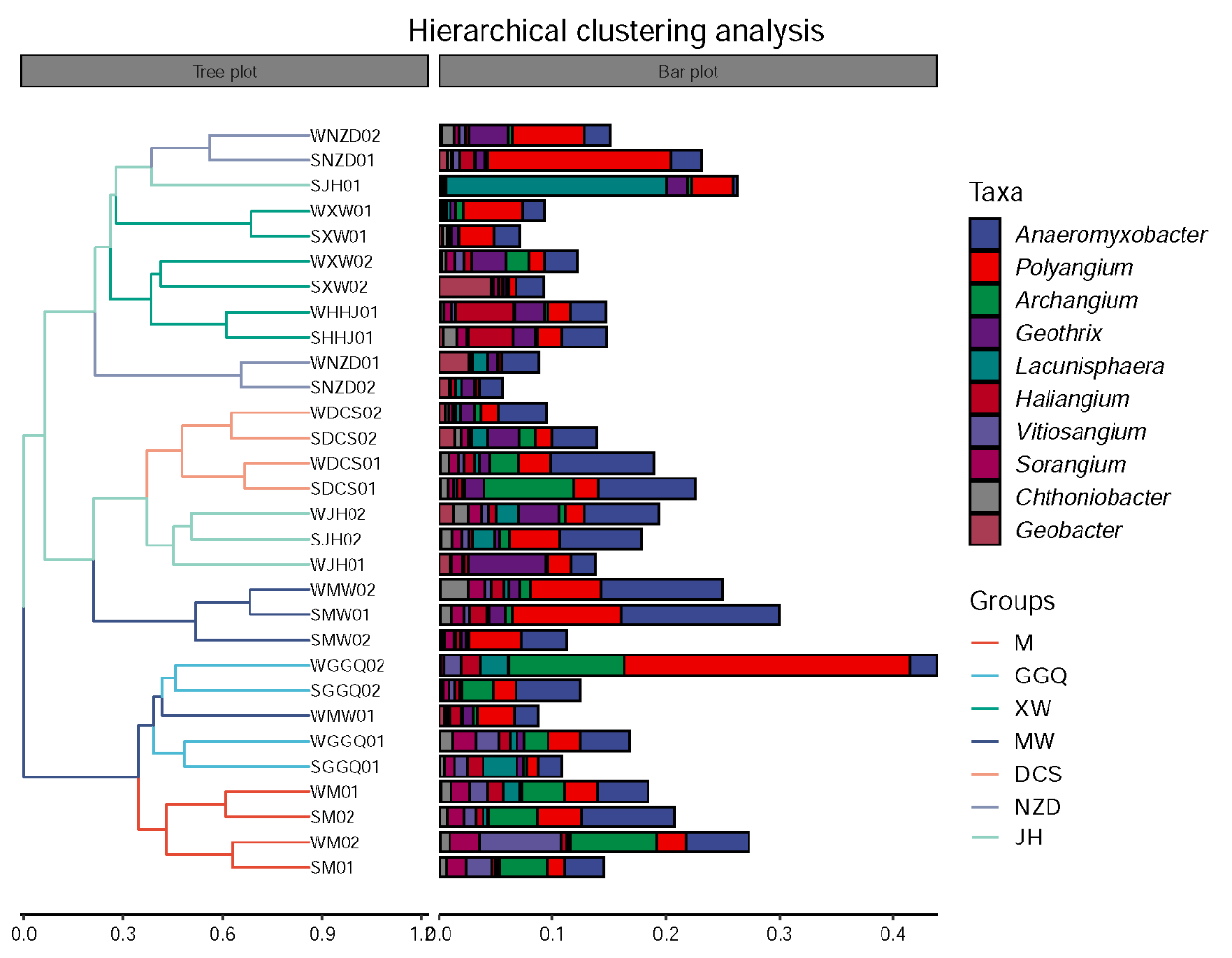
**

**Supplementary Figure S2 UPGMA clustering analysis of DNRA bacterial communities in sediments of cascade reservoir.**

**
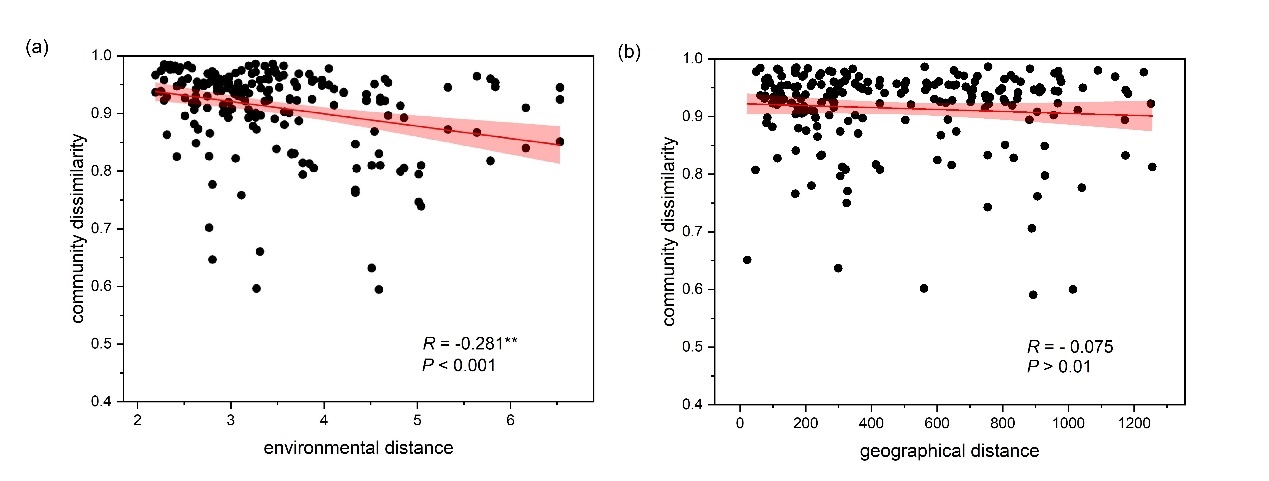
**

**Supplementary Figure S3 Relationship between geographic distance (a), environmental distance (b), and Bray-Curtis similarity of bacterial communities.**

**
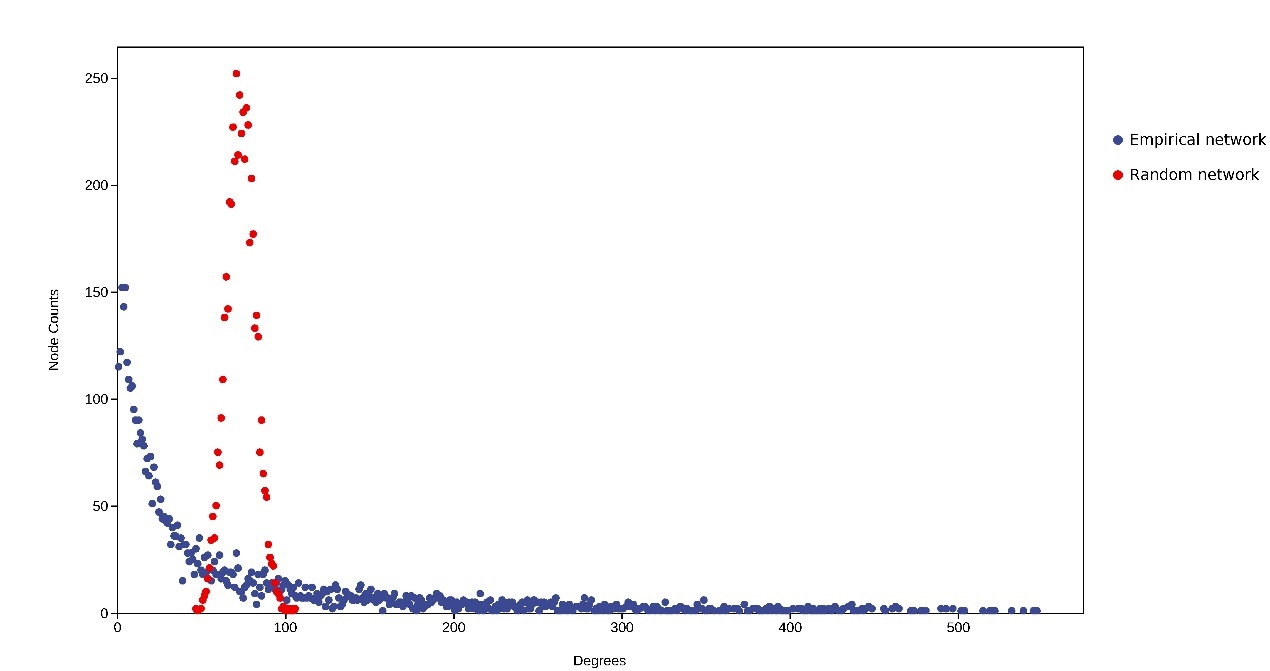
**

**Supplementary Figure S4. Different distributions of degree (number of connections) in observed co-occurrence network and a randomly generated Empirical network.**
